# Serum stress responsive gene EhslncRNA of *Entamoeba histolytica* is a novel long noncoding RNA

**DOI:** 10.1038/srep27476

**Published:** 2016-06-07

**Authors:** Arpita Saha, Sudha Bhattacharya, Alok Bhattacharya

**Affiliations:** 1 School of Life Sciences, Jawaharlal Nehru University, New Delhi, India; 2 School of Environmental Sciences, Jawaharlal Nehru University, New Delhi, India

## Abstract

Non coding RNAs are known to play important roles in regulating gene expression at the transcriptional and posttranscriptional levels in metazoans. There is very little information available about non coding RNAs in protists such as *Entamoeba histolytica*. Antisense and micro RNAs have been reported in *E. histolytica*, however no long non coding RNAs has been reported yet. Here, we report our findings on an *in vitro* serum stress-inducible gene EhslncRNA, a member of B1 transmembrane kinase family of *E. histolytica*. EhslncRNA encodes a transcript of 2.6 kb and sequence analysis revealed that there is no ORF >150 bp within this transcript. The transcript was found to be polyadenylated and mainly associated with monosomes in the cytoplasm under serum starvation. In normal proliferating cells this RNA is mainly present in the nucleus. The promoter element was mapped between 437 to 346 nucleotides upstream of transcriptional start site and has both positive and negative regulatory elements. Deletion of the negative element converted the promoter to serum inducible type. Oxygen and heat stress also increased expression levels of EhslncRNA. These observations suggest that EhslncRNA may be a long non coding RNA and likely to help cells withstand stressful conditions in the host.

*Entamoeba histolytica* is an important human pathogen infecting large number of people mainly from developing countries. Majority of the infected people do not show overt symptoms of amoebiasis. Among several molecules implicated in amoebic pathogenesis, transmembrane kinases (TMKs) appear to be one of the key molecules playing an important role in the disease pathology. Transmembrane kinases of *E. histolytica* were first characterized by Beck *et al.* using genomic data and bioinformatics analysis. These were further divided into nine sub groups (A, B1-3, C, D1-2, E and F) on the basis of signature motifs in the kinase domains and cysteine motifs in the extracellular domain^1^. B1 family of the TMKs has 35 members and EhTMKB1-9 was found to be a functional kinase and was mainly expressed in proliferating cells. Further analysis showed that the expression of EhTMKB1-9 is regulated by serum^2^. While searching for TMK genes expressed during proliferation, we identified EhTMKB1-18 as one of the genes that is induced in response to serum starvation^2^. EhTMKB1-18 expression is stimulated under serum starvation. Initial bioinformatics analysis of potential coding sequences suggested that this gene is unlikely to code for a protein due to lack of an open reading frame of significant size^2^. Therefore, it is likely to be a noncoding RNA that may be involved in stress response.

Short non coding regulatory RNAs have been described in *E. histolytica*^3^,^4^,^5^,^6^. These include small antisense RNAs and miRNAs. However, there are no reports of long non coding RNA (LNCR) molecules in *E. histolytica*. LNCRs play important role in the regulation of gene expression by functioning via *cis* and *trans* mode. These molecules have been found to affect various cellular processes ranging from cellular differentiation to cell cycle^7^,^8^. LNCRs have also been found to play important roles during both biotic and abiotic stress responses^9^ and during development (H19)^10^. Stress response appears to be an important function of LNCR. For example, growth arrest-specific transcript (GAS 5) stabilizes and functions as LNCR during serum starvation^11^ and during serum stress in mammalian systems. LNCRs have been found to affect cellular proliferation by modifying the chromatin signature^12^. Stress related LNCRs have been found to play important roles in coordinating different cellular networks to maintain cellular homeostasis or cell death^9^.

In this report, we have presented our results regarding characterization of EhTMKB1-18 transcript including tentative mapping of the promoter that is responsible for serum starvation response. In view of the functional role we have renamed EhTMKB1-18 as EhslncRNA (serum stress responsive long non coding RNA of *Entamoeba histolytica*). This is the first report of a starvation response gene regulatory system in the parasite *E. histolytica*. Our results also suggest a likely role of this stress induced non coding RNA in amoebic biology.

## Results

### Serum starvation induced gene EhslncRNA

**Figure 1:**
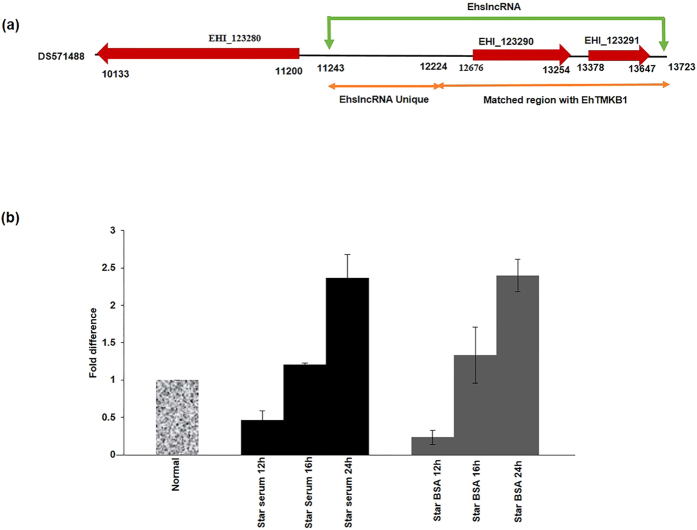
Time course of EhslncRNA expression on serum starvation. (**a**) Diagrammatic representation of contig DS571488. Annotated genes (EHI_123290 and EHI_123291) and upstream gene EHI_123280 are marked with red arrows representing the direction of transcription of the putative annotated genes. Green arrows indicate the start and the end of the non coding transcript of EhslncRNA. Orange arrows indicate region unique to EhslncRNA and matched region with EhTMKB1 respectively. (**b**) Quantitative real-time PCR was performed for measuring the level of EhslncRNA and RNA polymerase II gene transcripts using cells treated under different conditions as indicated. All samples were analyzed in triplicates, in three independent experiments. Values are normalized to the endogenous control (RNA Pol II) and results are expressed as percent fold change in comparison to normal proliferating cells (taken as 100%). BSA (0.5 mg/ml) was added to the cells during serum starvation.

The genomic map of EhslncRNA is shown in Fig. 1a. The transcript of this gene is made up of two annotated genes EHI_123290 (579 bp) and EHI_123291 (261 bp) (Fig. 1a). We believe that the transcript is much bigger (2.6 kb) than the two genes based on hybridization of different probes designed from different regions^2^. ORF analysis of the transcript displayed multiple stop codons in all reading frames (see supplementary Figure S1). The 5′ end of the transcript is likely to be a novel gene as no known homolog was found on bioinformatic analysis. However, the remaining 1.7 kbp towards the 3′ end displayed high degree of sequence identity with other EhTMKB1 members^2^. The gene was found to be evolutionary conserved. In all isolates/species stop codons were found to be conserved suggesting that the accumulation of stop codons is not recent (see supplementary Figures S2 and S3).

Analysis of EhslncRNA expression was carried out under increasing duration of starvation (Fig. 1b). The results clearly showed that there is a starvation time dependent increase in the expression. Increase in expression was observed from 16 h as compared to normal proliferating cells. It was not possible for us to increase starvation time beyond 24 h as cell viability decreased substantially after 24 h of starvation. Similar results were also observed when starvation was carried out in presence of low level of BSA. Based on these results we have carried out all starvation experiments for 24 h.

### EhslncRNA is polyadenylated and localized mainly in the cytoplasm in serum starved condition

**Figure 2:**
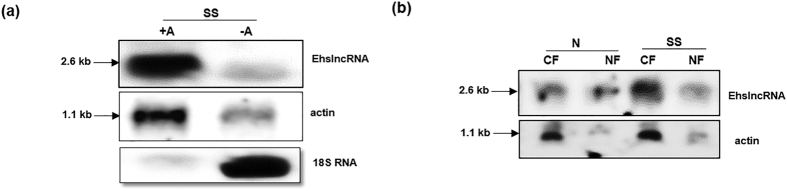
Localization and polyadenylation status of EhslncRNA. (**a**) Poly A^+^ and Poly A^−^ RNA was isolated from cells starved for 24 h and northern analysis was done using EhslncRNA specific probe. The blot was reprobed with actin as a loading control. 18S RNA as a control to check the quality of the poly A^+^ RNA preparation. SS, total RNA isolated from cells starved of serum for 24 h. (**b**) Nuclear and cytoplasmic RNA was isolated from normal cells and serum starved cells and northern analysis was done with EhslncRNA specific probe and blot was reprobed with actin taken as loading control. N, total RNA isolated from normal proliferating cells; SS, total RNA isolated from cells starved of serum for 24 h; CF, RNA isolated from cytoplasmic fraction; NF, RNA isolated from nuclear fraction.

In order to characterize EhslncRNA, we checked if this RNA is polyadenylated. This was done by northern analysis using EhslncRNA specific probe and poly A^+^ and poly A^−^ RNA fractions, isolated from serum starved cells. These results clearly showed that the EhslncRNA transcript is predominantly present in the poly A^+^ fraction of the RNA similar to that seen for actin transcripts (Fig. 2a). Ribosomal 18 S RNA was mainly found in the poly A^−^ fraction of total RNA as expected.

Localization of the EhslncRNA transcript was also investigated as many LNCRs are known to be present in the nucleus. Northern analysis with RNA isolated from nucleus and cytoplasm of normal proliferating cells and cells starved for 24 h, was performed. We observed that EhslncRNA is present in both nucleus and cytoplasm in normal proliferating cells. However, when cells were starved for 24 h, EhslncRNA was predominantly found in the cytoplasm as compared to that in the nucleus (Fig. 2b). The pattern was found to be similar to that seen for actin. These results suggest that EhslncRNA is polyadenylated and present both in nucleus and cytoplasm, but mainly in the later compartment in serum starved condition.

### EhslncRNA transcript is mainly present in the monosomes in serum starved condition

**Figure 3:**
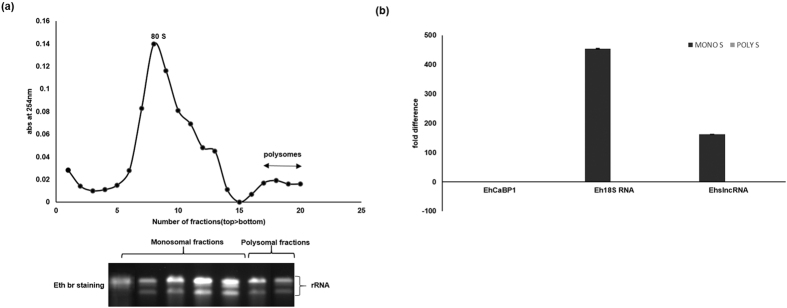
Localization of EhslncRNA in polysomes. **(a**) Upper panel shows the polysome profile of cells after serum starvation for 24 h. The lower panel shows the RNA isolated from 10–50% sucrose gradient fractions (monosomes and polysomes) and separated on 1.2% agarose-formaldehyde gel and visualized with ethidium bromide staining (Eth Br). **(b**) Quantitative real-time PCR was performed for measuring levels of EhslncRNA from ribosomes. Eh18SRNA and EhCaBP1 mRNA were extracted from monosomal and polysomal fractions isolated from serum starved condition as indicated. Values are normalized with EhCaBP1 in polysomal fractions of serum starved cells and results are expressed as percent fold change.

EhslncRNA transcript did not display any significant ORF, a feature present in the functional protein coding genes. It is possible that this RNA can still form polysomes and there may be a possibility that the small ORFs can actually be coding for functional peptides as seen in a few other systems^13^,^14^,^15^. Ribosomal profiling was carried out using total ribosomes taken from the normal proliferating cells and cells starved for 24 h without serum as described in details in “Methods” (Fig. 3a). The monosomal as well as polysomal fractions were pooled and then RNA was isolated from each of these two pooled fractions. Levels of EhslncRNA transcript was estimated by RT-PCR. Results showed that EhslncRNA is present in both monosomes and polysomes in the normal proliferating cells, however in the serum starved cells EhslncRNA is significantly higher in monosomes than in polysomes (Fig. 3b). The presence of EhCaBP1 mRNA and 18 S rRNA were used to assess the quality of polysomal/monosomal fractions respectively. Presence of the EhslncRNA transcript in polysomes does indicate that there is a possibility of the expression of small peptides under normal conditions.

### Promoter analysis of EhslncRNA

**Figure 4:**
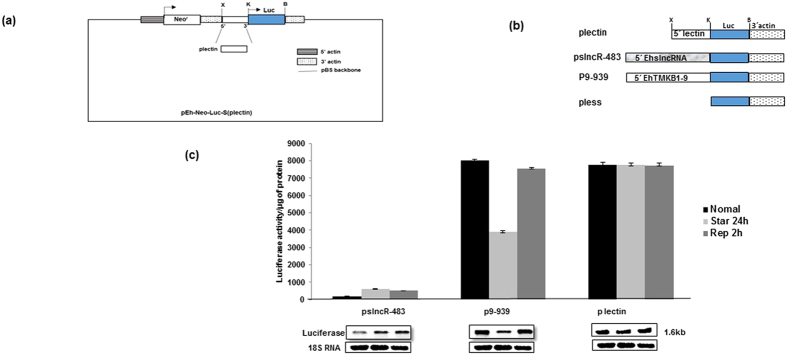
Promoter mapping of EhslncRNAusing luciferase reporter gene. (**a**) Diagrammatic representation of reporter luciferase vector pEh-Neo-Luc-S (plectin). X, K, B are *Xho*I, *Kpn*I and *BamH*I sites respectively and Luc represents luciferase gene. (**b**) Schematic representation of the different promoter fragments cloned in the pEh-Neo-Luc-S (plectin). These fragments were cloned in *Xho*I and *Kpn*I sites. (**c**) Luciferase expression was determined by measuring the reporter enzyme activity. Stable transfectants of the indicated constructs were used and each of these cell lines was subjected to three different conditions – normal proliferation, serum starvation for 24 h and replenishment for 2 h after serum starvation.

EhslncRNA transcript is made up of two annotated genes EHI_123290 and EHI_123291 and the transcription start point (TSP) of this gene is about 40 nucleotides downstream of the next gene (EHI_123280) suggesting that the promoter may lie partly within the upstream coding gene^2^. Therefore, the promoter of EhslncRNA was mapped by cloning the upstream fragment (−437 to +45) of the gene in a luciferase reporter gene vector (pEh-Neo-luc-S, plectin) as described in “Material and Methods” and schematically shown in Fig. 4a. For comparison, a luciferase construct of EhTMKB1-9 containing around 1000 nucleotides upstream from the start codon was used. A promoter-less vector (pless) was constructed in which the complete promoter region of the parent vector was removed and taken as a negative control (Fig. 4b). These constructs were transfected in trophozoites and the transformed cells were selected using the drug G418. The strength of the promoters was determined by luciferase activity and the construct pslncR-483 showed around 200 fold higher activity as compared to pless construct. However, the activity was much less compared to that using plectin and p9-939 promoters under proliferative conditions (Fig. 4c). It was inferred from this observation that the pslncR-483 is a weaker promoter as compared to the promoter of EhTMKB1-9. These stable transfectants were then subjected to starvation for 24 h followed by replenishment with serum for 2 h. Luciferase values for pslncR-483 was high (3 fold) on serum starvation and remained unchanged after 2 h in presence of serum similar to that seen with endogenous gene. Northern analysis was used to confirm the change in transcript levels (Fig. 4c) and the results showed the same pattern. All these results indicate that the promoter of EhslncRNA is regulated by serum starvation.

### Mapping the transcription regulatory region of EhslncRNA

**Figure 5:**
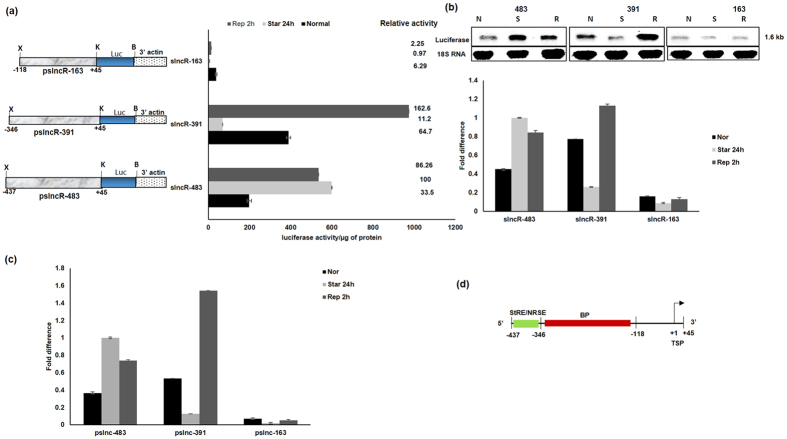
Deletion mapping of EhslncRNA upstream region. (**a**) Schematic representation of EhslncRNA deletion constructs containing upstream sequences with indicated genomic positions that were cloned upstream of luciferase (luc) gene. X, K, B are *Xho*I, *Kpn*I, *BamH*I sites respectively. Reporter luciferase activity of the stable transfectants under different conditions- normal proliferation, serum starvation 24 h and replenishment for 2 h after starvation. Activity of pslncR-483 under starvation condition is taken as 100 for relative fold calculation. (**b**) Stable transfectants of the indicated constructs were used and each of these cell lines was subjected to three different conditions – normal proliferation, serum starvation for 24 h and replenishment for 2 h after serum starvation. Total RNA was isolated and northern analysis was carried out using luciferase specific probe. The blot was reprobed with 18SRNA as loading control. Fold difference was calculated with respect to pslncR-483 construct under starvation condition and taken as 1 for fold calculation. (**c**) Quantitative real-time PCR was performed for measuring the level of luciferase gene and RNA polymerase II gene transcripts in different promoter deletion constructs of EhslncRNA and treated under different conditions as indicated in the cells. All samples were analyzed in triplicates, in three independent experiments. Values are normalized to the endogenous control (RNA Pol II) and results are expressed as percent fold change in comparison to pslncR-483 construct under starvation condition (taken as 100%). (**d**) Schematic representation of the promoter region of EhslncRNA. Arrow indicates transcription start point (TSP). Green box refers to starvation responsive element (StRE)/negative repressor of serum element (NRSE). Red box indicates basal promoter (BP) region.

Different promoter constructs were made by deletion and their activity on starvation was determined in order to identify the starvation response element. Details about different constructs are shown in Fig. 5a. *E. histolytica* cells were then transfected to generate stable cell lines and reporter luciferase assays were performed using these cells. The deletion construct pslncR-391 (comprising region from −346 to +45) displayed serum dependent expression, and not starvation inducible expression. We observed a decrease in expression on serum starvation and a significant increase on serum replenishment. However, the construct pslncR-163 (comprising region −118 to +33) gave a pattern similar to pslncR-391, but with very low level of expression. It appears that this deletion also removes a part of the main promoter along with starvation inducible promoter. Since some activity was still observed, though very low, it is possible that a part of basal promoter may still be present in this construct. Our results suggest that the starvation responsive region lies between −437 to −346 (Fig. 5b,c). The region between −437 to −346 acts as negative repressor of serum response and overall the organization of EhslncRNA promoter is shown in Fig. 5d.

### EhslncRNA acts as the stress regulator

**Figure 6:**
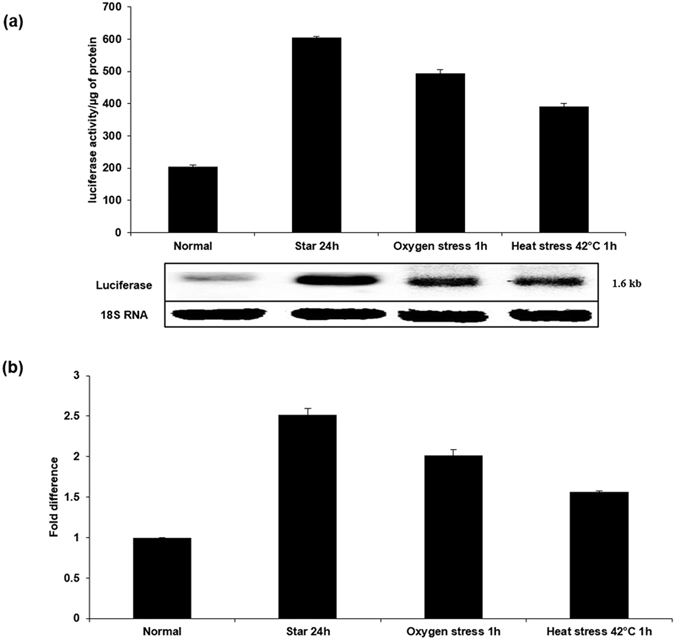
Expression of EhslncRNA under different stress conditions. (**a**) Reporter luciferase activity of the stable transfectant pslncR-483 was used for checking the effect of different stresses as indicated. Luciferase gene expression was also determined by northern analysis using luciferase gene as probe. Cells containing pslncR-483 were subjected to different conditions – normal proliferation, serum starvation for 24 h, oxygen stress for 1 h and heat stress at 42 °C for 1 h. The blot was reprobed with 18S RNA as loading control. (**b**) Quantitative real-time PCR was performed for measuring levels of EhslncRNA and RNA polymerase II gene transcripts in samples from cells treated under different conditions as indicated. All samples were analyzed in triplicates, in three independent experiments. Values are normalized to the endogenous control (RNA Pol II) and results are expressed as percent fold change in comparison to normal proliferating cells (taken as 100%).

When the microbial pathogens enter the host it is exposed to different types of stresses, such as temperature, oxygen and pH that lead to expression of a set of genes required to cope with stress^16^. Many microorganisms have evolved master regulators, such as alternative sigma factors to coordinate the expression of multiple loci required for adaptation to environmental and/or physiological stress^17^,^18^,^19^. In general, many of the stress response genes are not specific for a given stress, but are pleotropic meaning; that these get activated in presence of any stress. In order to test this, EhslncRNA expression was determined after amoebic cells were subjected to different types of stresses, such as heat (42 °C for 1 h) and oxygen (aeration). Real time PCR and reporter luciferase assay were carried out using cells containing pslncRNA-483. The results showed that oxygen stress and high temperature increased the expression of EhslncRNA, similar to that seen for serum starvation (Fig. 6a,b). We inferred from these observations that the EhslncRNA may be acting as a general stress regulator.

## Discussion

Living organisms when challenged with different types of stresses employ elaborate mechanisms to overcome these responses. Parasites reside in unique ecological niche of the host and are exposed to different type of stresses, such as oxygen limitation or excess, restriction of food, high temperature and pH. Therefore, these parasites have developed adaptive mechanisms for survival under adverse conditions. Usually the mechanisms involve modifications at different levels; transcriptional, translational and post translational modifications. We have explored the properties of a potential non coding transcript, EhslncRNA which is highly expressed under serum stress and other stress conditions in *E. histolytica* and the results have been presented in this report. Together with our previous studies, we conclude that EhslncRNA plays an important role in the amoebic stress response. This transcript is similar to many other non-coding transcripts that have been implicated in stress response^9^,^10^,^11^,^20^,^21^,^22^,^23^.

We are not sure about the reason for a decrease in the expression of EhslncRNA after 12 hours of serum starvation. We can speculate that after a few hours of starvation, in general, metabolic activity decreases and a reduction in transcription may be an effect of that. It is also likely that after a few hours of starvation degradation of RNA and other components may be taking place in order to maintain metabolic pool. We also believe that the enhanced transcription activity at later time points may be due to adaptive process that helps cells to cope with stress. Once adapted to starvation, the transcriptional machinery may get selectively activated and only a handful of genes including EhslncRNA, is reactivated after 16 h onwards of serum starvation till 24 h.

Results indicated that this non coding transcript is polyadenylated similar to many long non coding RNAs transcribed by RNA polymerase II^24^. This transcript has very low coding potential as it does not encode a significant ORF, however the presence of smaller functional ORFs cannot be ruled out completely. There are a few indicators that suggest small peptide coding potential of EhslncRNA transcript. The main one is its association with polysomes. This has been seen in other long coding RNAs that are known to encode small peptides^25^,^26^,^27^. Moreover this RNA is present in the cytoplasm^28^, similar to other peptide encoding large noncoding RNAs^29^,^30^,^31^. EhslncRNA transcript is also present in the nucleus in normal proliferating cells of *E. histolytica*, however it accumulates in the cytoplasm under serum starved conditions. It appears that this transcript may have roles both in nucleus and cytoplasm in normal cells.

Putative promoter elements and genome organization of *E. histolytica* was found to be distinct from metazoans and better characterized protozoan organisms, such as *Leishmania* and *Plasmodium*. Expression of EhslncRNA on serum starvation is regulated at transcriptional level. Deletion analysis of EhslncRNA promoter clearly mapped the starvation responsive region between −437 to −346. We also mapped the region responsible for serum-induced response from −346 to −118. This region also carries a part of the basal promoter. We believe that the overall organization of the promoter element includes multiple elements comprising of serum inducible, serum repressible and basal promoter. Interaction of these elements with each other are responsible for overall property of this gene.

Organisms require certain environment for its growth and function. Various mechanisms have evolved to help organisms survive in face of harsh and adverse external conditions of the environment. Multicellular organisms, due to defined division of labor, utilize designated organs and tissues to provide a relatively stable and homogenous internal environment. Unicellular organisms, such as yeast *S. cerevisiae* have evolved autonomous mechanisms for adapting to drastic environmental changes. There are number of genes that are transcribed in response to a variety of stresses and have been found to be responsible for general yeast stress response^32^,^33^. These genes are regulated by shared promoter elements utilized by common transcription factors^34^. The results presented here also show that EhslncRNA is acting as general stress regulator utilizing a unique promoter with modular elements with diverse stress response property.

## Methods

### Growth conditions and maintenance of cell culture

Trophozoites of *E. histolytica* strain HM-1:IMSS(clone 6) were used to carry out all the experiments. The cells were maintained in TYI-33 medium supplemented with 15% adult bovine serum, 1X diamond’s vitamin mix and antibiotics (0.3 units/ml penicillin and 0.25 mg/ml streptomycin) at 35.5°C 35. Serum starvation conditions were achieved by replacing the TYI-33 medium supplemented with serum from mid log phase grown Entamoeba with TYI-33 medium containing 0.5% adult bovine serum for indicated time period. Serum replenishment was achieved by decanting the medium after 24 h of serum starvation with indicated compounds for 2 h. G-418 (Sigma) was added at 10 μg/ml to maintain the cell lines.

### Transfection and selection of *E. histolytica* trophozoites

Transfection was done by electroporation as described previously^36^. Briefly, trophozoites in log phase were harvested and washed with Phosphate-buffered saline (PBS) followed by incomplete cytomix buffer (10 mM K_2_HPO_4_/KH_2_PO_4_ (pH 7.6), 120 mM KCl, 0.15 mM CaCl_2_, 25 mM HEPES (pH 7.4), 2 mM EGTA, 5 mM MgCl_2_). The washed cells were then re-suspended in 0.8 ml of complete cytomix buffer (incomplete cytomix containing 4 mM adenosine triphosphate, 10 mM glutathione) containing 200 mg of plasmid DNA and subjected to two consecutive pulses of 3000 V/cm (1.2 kV) at 25 mF (Bio-Rad, electroporator). The transfectants were initially allowed to grow without any selection. Drug selection was initiated after 2 days of transfection in the presence of 10 mg/ml G-418 for constructs with luciferase reporter gene.

### Luciferase reporter constructs

EhslncRNA promoter fragment −437 to +45 was cloned in p lectin by excising the 5′-lectin promoter region with *Xho*I and *Kpn*I and was replaced with PCR product obtained using the EhslncR 437 FP 5′CGGCTCGAGAAATTAATGAAATTAGAGAAATTAGG3′ and EhslncR 45 RP 5′CGCGGGTACCAATTAGTTTATTCATACTTAT 3′. p9-939 and p less construct were already cloned in the p lectin vector^2^. All deletion constructs were PCR amplified with *Xho*I site in the forward primer at the desired position and *Kpn I site* in the reverse primer. All the fragments were PCR amplified from genomic DNA and inserted at *Xho*I and *Kpn*I sites upstream of luciferase gene. The orientation and sequence of each construct was confirmed by restriction digestion and DNA sequence analysis. The *Xho*I and *Kpn*I sites are underlined in the primers. List of the primers and the cloning done in this study are provided in the Tables 1 and 2 respectively in supplementary information.

### Luciferase Assay

The protocol was performed as described previously^37^. In short, stably transfected trophozoites, maintained in TYI-S-33 medium supplemented with 10 mg/ml G-418, were chilled on ice, harvested and washed once in PBS (pH 7.4), and lysed in 200 ml of reporter lysis buffer (Promega) with the addition of protease inhibitor cocktail (Sigma). Lysates were frozen overnight at −80 °C. After thawing on ice for 10 min, cellular debris was pelleted, and the samples were allowed to warm to room temperature. Luciferase activity was measured according to the manufacturer’s instructions (Promega) using a Glomax 20/20 Luminometer. Luciferase activity per mg of protein was calculated as a measure of reporter gene expression.

### RNA isolation and Northern hybridization

Total RNA from ~5 × 10^6^ cells was isolated using Trizol (Invitrogen) as per manufacturer’s instructions. Poly A^+^ and PolyA^−^ RNA fractions were obtained using polyATtract mRNA isolation system (Promega) as per protocol prescribed by manufacturer. Nuclear and cytoplasmic RNA were fractionated following protocol from^38^. Briefly, cells were washed with pre-chilled PBS and then cells were pelleted down at 1000 × g for 5 min at 4 °C. Pelleted cells were then resuspended in cold cell disruption buffer [1.5 M MgCl_2_, 10 mM KCl, 20 mM Tris Cl (pH 8.0), 1 mM DTT] and incubated for 10 min in ice. The cell membranes were disrupted by glass dounce homogenizer. Homogenate were transferred to new microfuge tube in which 10% Triton X-100 (sigma) were added to a final concentration of 0.1%. The tube was inverted several times to mix properly. The nuclear and cytoplasmic fraction was isolated by centrifuging the homogenate at 1500 g for 5 minutes at 4 °C. RNA samples (30 μg) were resolved in formaldehyde agarose in gel running buffer [0.1 M MOPS (pH 7.0), 40 mM sodium acetate, 5 mM EDTA (pH 8.0)] and 37% formaldehyde at 4 V/cm. The RNA was transferred on to GeneScreen plus (NEN) nylon membranes. Probe was prepared by random priming method using NEBlot kit (NEB). Hybridization and washing conditions for RNA blots were as per manufacturer’s protocol.

### Quantitative Real Time (qRT-PCR)

Real time PCR efficiencies for each gene were calculated from the slope, according to the established equation E = 10^[−1/slope]^ using genomic DNA as template (serial 1:10 fold dilutions) and were found around 1.96 ± 0.06^39^. Two μg of total RNA (DNase I treated) was reverse transcribed using random hexamers into cDNA by Superscript III reverse transcriptase (Invitrogen). Real time quantitative PCR was performed in 7500 Real Time PCR System (Applied Biosystems) using SYBR green PCR Master Mix, 2 pmol of forward and reverse primers and 2 μl of cDNA (serial 1:10 fold dilution). EhslncRNA, EhCaBP1, 18 S RNA, Ehluc and the RNA Pol II (control gene) were amplified in parallel. The conditions were predenaturation at 95 °C for 10 min, followed by 40 cycles at 95 °C for 15 sec and 58 °C for 1 min followed by a dissociation stage at 95 °C for 15 sec and 58 °C for 1 min. Cycle threshold values (Ct) were analyzed by the SDS1.4 software (Applied Biosystems) and all samples were analyzed in triplicates in three independent experiments. Reactions without cDNA were used as no template control and no RT controls were also set up to rule out genomic DNA contamination. Relative quantification of EhTMKB1expression was determined using the comparative Ct method (ABI Prism 7500, SDS User Bulletin; Applied Biosystems).

### Polysome Isolation

Polysomes were isolated from *E. histolytica* trophozoites by using the combination of protocols which was modified a little^40^,^41^. Briefly, 200 μM cycloheximide was added to cells for10 min at 37 °C. Cells were pelleted for 5 min at 500 × g and washed twice with pre-chilled PBS containing 200 μM cycloheximide. Pellet were kept on ice and were subsequently lysed by lysis buffer in (1% (v/v) Igepal CA-360 and 0.5% (w/v) sodium deoxycholate in polysome buffer [400 mM potassium acetate, 25 mM potassium HEPES (pH 7.2), 15 mM magnesium acetate, 200 μM cycloheximide, 1 mM dithiothreitol (DTT)] to make a final volume of 3 ml. After a 10 minute incubation on ice, lysates were centrifuged for 10 minutes at 20,000 × g at 4 °C. 3 ml of the clarified lysate were then loaded on top of 1 ml sucrose cushion (1 M sucrose in polysome buffer) in 4 ml polyallomer ultracentrifuge tubes and then centrifuged for 123 minutes at 50,000 rpm at 4 °C in an SW 55 Ti rotor (BeckmanCoulter) to concentrate the ribosomes. Ribosome pellets were then resuspended in 500 μl of polysome buffer, incubated for at least 30 minutes at 4 °C for complete ribosome resuspension and centrifuged for 10 minutes at 12,000 × g at 4 °C. The ribosome suspension was finally layered on top of a 3 ml continuous linear 10 to 50% sucrose (w/v) gradient in polysome buffer and centrifuged for 1.5 h at 50,000 rpm at 4 °C in an SW 55 Ti rotor. Fractions of 330 μl were collected manually and absorbance taken at 254 nm using NanoDrop 2000c spectrophotometer (Thermo Scientific). Polysome fractions were digested with 200 μg Proteinase K (Sigma) for 1 h at 37 °C. RNA was extracted with acid phenol:chloroform:isoamylalcohol, pH 4.5 (Life Technologies), extracted twice with chloroform and then precipitated using isopropanol.

### Sequence alignment and ORF prediction

ORF prediction of EhslncRNA gene was done using Bioedit sequence alignment editor (version 7.0; Tom Hall). Genome sequences of *Entamoeba histolytica* strains and Entamoeba species was extracted from amoebadb (http://amoebadb.org/amoeba/) into the Bioedit sequence alignment editor. Multiple sequence alignment was done using Clustal omega (http://www.ebi.ac.uk/Tools/msa/clustalo/).

## Additional Information

**How to cite this article**: Saha, A. *et al.* Serum stress responsive gene EhslncRNA of *Entamoeba histolytica* is a novel long noncoding RNA. *Sci. Rep.* **6**, 27476; doi: 10.1038/srep27476 (2016).

## Supplementary Material

Supplementary Information
