# Supplementary material for: Serum stress responsive gene EhslncRNA of Entamoeba histolytica is a novel long noncoding RNA
Source: Sci Rep. 2016 Jun 7;6:27476. doi: 10.1038/srep27476 (PMC4895391; doi:10.1038/srep27476)
Supplement: Supplementary Information [file srep27476-s1.pdf]

Supplementary information

Serum stress responsive gene EhslncRNA of *Entamoeba histolytica* is a novel long non coding RNA

Arpita Saha[1], Sudha Bhattacharya[2], Alok Bhattacharya[1]

[1] School of Life Sciences, Jawaharlal Nehru University, New Delhi, India

[2] School of Environmental Sciences, Jawaharlal Nehru University, New Delhi, India

>gb|DS571171.1|:11243-13723 Entamoeba histolytica HM-1:IMSS scf_1104750517413 genomic scaffold, whole genome shotgun sequence Frame 1

1 AGA GAT TTA AAA AAT AAA TGA TAT GAT GTC TAA ATA AAA ATA GAA 45
1 Arg Asp Leu Lys Asn Lys End Tyr Asp Val End Ile Lys Ile Glu 15

46 AAT TGA TAT TTT ATT TTT ATG AAA ATA ACG AGT ATA ATC TAT GAG 90
16 Asn End Tyr Phe Ile Phe Met Lys Ile Thr Ser Ile Ile Tyr Glu 30

91 ATT ATT CAT TTC ATT ATT GTC AAT TTT ATT ATT AAT GTC AAA CCA 135
31 Ile Ile His Phe Ile Ile Val Asn Phe Ile Ile Asn Val Lys Pro 45

136 CAC TTT ATG ACA GAA GAA GTA ATT TTT TCA TTA AAT TAA ACT AAT 180
46 His Phe Met Thr Glu Glu Val Ile Phe Ser Leu Asn End Thr Asn 60

181 GTT AAA TCC TCG ATA AAT TAA TTT TTA ATT TCC TAA GTC AAT ATA 225
61 Val Lys Ser Ser Ile Asn End Phe Leu Ile Ser End Val Asn Ile 75

226 ATA AAT ATT CTT CAT TTT GGT GAT ATG AGA AGA AAT TAT ACA TTT 270
76 Ile Asn Ile Leu His Phe Gly Asp Met Arg Arg Asn Tyr Thr Phe 90

271 TTT AAG AAG ATT GGT AAA CGC CAT TAT GTG ATG AAT AGT ACA TTT 315
91 Phe Lys Lys Ile Gly Lys Arg His Tyr Val Met Asn Ser Thr Phe 105

316 TAA AAC TTT ATG TCT TTC AAT TTC TGT TAG TCA GTT AAG TGA AAC 360
106 End Asn Phe Met Ser Phe Asn Phe Cys End Ser Val Lys End Asn 120

361 AAT GTT ATT TTA TAA ATT AAT ATT ATT TTC TAT AAG ATA TTG CCA 405
121 Asn Val Ile Leu End Ile Asn Ile Ile Phe Tyr Lys Ile Leu Pro 135

406 TAT TAA TAA ACA ATG GAA TTT TCA TTT TAT TGA ACT TTA AGA GAA 450
136 Tyr EndEnd Thr Met Glu Phe Ser Phe Tyr End Thr Leu Arg Glu 150

451 CAA AGA TTA ATT AGA GTA TAT GTA TTT AAA AGA CTA AAT GTT GGT 495
151 Gln Arg Leu Ile Arg Val Tyr Val Phe Lys Arg Leu Asn Val Gly 165

496 GGT AAG AGT TTT TTA GAA AAG AGT TAA TTA ACT CAA AGG TAT TGA 540
166 Gly Lys Ser Phe Leu Glu Lys Ser End Leu Thr Gln Arg Tyr End 180

541 AGA AAG AAG TGT TTT GAA TAA CTT CAT TGT ATT GGT AGT TAA TTA 585
181 Arg Lys Lys Cys Phe Glu End Leu His Cys Ile Gly Ser End Leu 195

586 AAA TGA AAT AAA AGA CTC TCG TTA TAA GTA GGA AGA TGA AGA GCA 630
196 Lys End Asn Lys Arg Leu Ser Leu End Val Gly Arg End Arg Ala 210

631 ACA TTC ATT AGA AAA ATT GAT TTC ATT GCA TAA TAT TAT TTA TTG 675
211 Thr Phe Ile Arg Lys Ile Asp Phe Ile Ala End Tyr Tyr Leu Leu 225

676 AAT AAT TTT TTA ATT CTT CTA TCA TAA AAA ATA CAA AAA AAT GAA 720
226 Asn Asn Phe Leu Ile Leu Leu Ser End Lys Ile Gln Lys Asn Glu 240

721 TAA ATT CAA TAA TAT AAT AAA TCT CAT TTA GAA AAA GTA TAG TGC 765
241 End Ile Gln End Tyr Asn Lys Ser His Leu Glu Lys Val End Cys 255

766 AGG AAC AAA ACG AGT ATT TCC ATT AAA ACC AAC AAA TGT CTG TGG 810
256 Arg Asn Lys Thr Ser Ile Ser Ile Lys Thr Asn Lys Cys Leu Trp 270

811 TTG TAT CAC TAA TTC TCT ACA CAT AGG ACA TCT TGG TTG TTG AAT 855
271 Leu Tyr His End Phe Ser Thr His Arg Thr Ser Trp Leu Leu Asn 285

856 AAG CCA TTT AAA AAT ACA TTC TTC ACA ATA AAC ATG ACC ACA TTT 900
286 Lys Pro Phe Lys Asn Thr Phe Phe Thr Ile Asn Met Thr Thr Phe 300

901 TAA CTT AAT TGG ACG ATT AAC TGT GTC TTG ACA AAT TAA GCA CAT 945
301 End Leu Asn Trp Thr Ile Asn Cys Val Leu Thr Asn End Ala His 315

946 TTT GTC TTC TAA TGA ATC TAC GGT TAC TGA AGT ACC AAA CAT ATT 990
316 Phe Val Phe EndEnd Ile Tyr Gly Tyr End Ser Thr Lys His Ile 330

991 GTT TTG AGT GAA AAA GGT TTT AAG AAC TTT AAA GAA ACA AGA AAG 1035
331 Val Leu Ser Glu Lys Gly Phe Lys Asn Phe Lys Glu Thr Arg Lys 345

1036 AAA ATT AAC AAA TCC AAG ACA ACG TTC AAA CAC ATA AAG CCA CGT 1080
346   Lys Ile Asn Lys Ser Lys Thr Thr Phe Lys His Ile Lys Pro Arg   360

1081 TAT TGG ATA TAT AAC AAT ATA ACT AAT CCA TTG GTT GGT TAT TGA 1125
361   Tyr Trp Ile Tyr Asn Asn Ile Thr Asn Pro Leu Val Gly Tyr End   375

1126 AGT GTA TGA AAT AAG ATA ATT ATA AAT AAT AAA AGT AGG AAG AAT 1170
376   Ser Val End Asn Lys Ile Ile Ile Asn Asn Lys Ser Arg Lys Asn   390

1171 AAC TCT TAT AGA CAA AGT AAG TGA TTC TAT CAT AGA GAG TAA TTT 1215
391   Asn Ser Tyr Arg Gln Ser Lys End Phe Tyr His Arg Glu End Phe   405

1216 ACA TAA TAA TAA TAT ATT TTT AAC TAA AAA TAT TGT TAA AAC TTT 1260
406   Thr EndEndEnd Tyr Ile Phe Asn End Lys Tyr Cys End Asn Phe   420

1261 TAG ATA ATA ATT TAT ATA ACG GTA AAT ACA GTC ACA TAG ATA TAA 1305
421   End Ile Ile Ile Tyr Ile Thr Val Asn Thr Val Thr End Ile End   435

1306 ATA AAT GAA TAA ATC ACT AAA AGA ATT TGG AAT AAC AGT AAT ATC 1350
436   Ile Asn Glu End Ile Thr Lys Arg Ile Trp Asn Asn Ser Asn Ile   450

1351 TAT CAT ATC AGT TAT TCC TAT TTG CCA CAC CTT TAT AAT AAA TAA 1395
451   Tyr His Ile Ser Tyr Ser Tyr Leu Pro His Leu Tyr Asn Lys End   465

1396 TAA CAT AGA AGG AAT ACA ATA AAC TAA TAT CCT TAT TTT ATT TTC 1440
466   End His Arg Arg Asn Thr Ile Asn End Tyr Pro Tyr Phe Ile Phe   480

1441 CCC AGT ATT TAC ATA TTG TGT AAC TAA CTC AGT TAA ATG ATA ATG 1485
481   Pro Ser Ile Tyr Ile Leu Cys Asn End Leu Ser End Met Ile Met   495

1486 TTG AAT GAA ATC AAC TAT AAT AAA TAA AGT ATA AAA AAT ATG ACT 1530
496   Leu Asn Glu Ile Asn Tyr Asn Lys End Ser Ile Lys Asn Met Thr   510

1531 AAA GAA AAA GAA TAA TAT AAC TAC TAT ATA AGA CCC AAA ACC TGA 1575

511 Lys Glu Lys Glu End Tyr Asn Tyr Tyr Ile Arg Pro Lys Thr End 525

1576 ATC ATA ATC ATT ATT TAT ATA ATC ATT ATC ACC ATC ATT ATT ATT 1620
526 Ile Ile Ile Ile Ile Tyr Ile Ile Ile Ile Thr Ile Ile Ile Ile 540

1621 ATT ATT TTG GTT GTT TGG TTG TTG TTC ATT TCC TGT ATG AGG TAC 1665
541 Ile Ile Leu Val Val Trp Leu Leu Phe Ile Ser Cys Met Arg Tyr 555

1666 TTC ATT TAA TTG AGT TAA ACC TTC TTC TCT CAA ATT AAC TTC ATG 1710
556 Phe Ile End Leu Ser End Thr Phe Phe Ser Gln Ile Asn Phe Met 570

1711 TTC ATC AAC ATT TCC CAT TTC AAT TTC GTG TTC AAA TTT TGT GTC 1755
571 Phe Ile Asn Ile Ser His Phe Asn Phe Val Phe Lys Phe Cys Val 585

1756 TTG GTT TTC ATT AGT ATT TAA TGA ATT TAT ATG ATT ATT TTC ATG 1800
586 Leu Val Phe Ile Ser Ile EndEnd Ile Tyr Met Ile Ile Phe Met 600

1801 AGT GTC TAT ACA ACT CAA TTT ATT TGT ACA GAC AAT ATC ACT ATC 1845
601 Ser Val Tyr Thr Thr Gln Phe Ile Cys Thr Asp Asn Ile Thr Ile 615

1846 ACA TTC TTT TTC TGT TTT CTG TTT ATT AAG ATG TTC TTC AGT TTG 1890
616 Thr Phe Phe Phe Cys Phe Leu Phe Ile Lys Met Phe Phe Ser Leu 630

1891 TGA CAT TTA AGT GAT TTT ATA GTA TTT TTT TGT AAA CTT TTT AGA 1935
631 End His Leu Ser Asp Phe Ile Val Phe Phe Cys Lys Leu Phe Arg 645

1936 AAT AAA AAA TGA GCA GAA ACA GAA TAT CAA AAA CCC TTT ATT ATT 1980
646 Asn Lys Lys End Ala Glu Thr Glu Tyr Gln Lys Pro Phe Ile Ile 660

1981 TAT TCT TAA TCA AAT CAA CCA TCT CCT CAA TTG TTA TCT CAT TAA 2025
661 Tyr Ser End Ser Asn Gln Pro Ser Pro Gln Leu Leu Ser His End 675

2026 ATG TGT GAA TTG CCA GGT TGC ATC CTT TAA TTT GAA GTA ATT CAT 2070
676 Met Cys Glu Leu Pro Gly Cys Ile Leu End Phe Glu Val Ile His 690

2071 TAA CAG TCA TTT CTG AGG CAA TTC CTA TTG GAG TAG CAC CAG CTG 2115
691 End Gln Ser Phe Leu Arg Gln Phe Leu Leu Glu End His Gln Leu 705

2116 ATA ATG CAG AAT GAA TAC CTG AAA TAG TAT CTT CAA ATA CAA TAC 2160
706 Ile Met Gln Asn Glu Tyr Leu Lys End Tyr Leu Gln Ile Gln Tyr 720

2161 AAT GAG AAA TAC TAA CTC CTA ATT TCT TAG AAG CTT TTA AAT AAA 2205
721 Asn Glu Lys Tyr End Leu Leu Ile Ser End Lys Leu Leu Asn Lys 735

2206 TGT CAG GAG CAG GTT TAC CTT TAA AAG TAA AAT CAT TAA AAA TAA 2250
736 Cys Gln Glu Gln Val Tyr Leu End Lys End Asn His End Lys End 750

2251 TTT TAT CAA TAT CAA ACC ATT CTT CCA AGT GGT ATT TTT GAA TAA 2295
751 Phe Tyr Gln Tyr Gln Thr Ile Leu Pro Ser Gly Ile Phe Glu End 765

2296 ACA CTT GAA CAT TTC CCC AAT CAC TTG AAG TGG CAA TAG TAA AAG 2340
766 Thr Leu Glu His Phe Pro Asn His Leu Lys Trp Gln EndEnd Lys 780

2341 GAA TAT TAC ATT TTT TTA ATG CCT CAA AAA GAT TAA TTG CAC CAT 2385
781 Glu Tyr Tyr Ile Phe Leu Met Pro Gln Lys Asp End Leu His His 795

2386 CAC ATA ATT TTA TAT CAC TTT TCA TTA ACA TAG TTC TAT ATA AAT 2430
796 His Ile Ile Leu Tyr His Phe Ser Leu Thr End Phe Tyr Ile Asn 810

2431 TTT CTT TTT CTT CAG CAT ATT TCT TTG CAT CTT CAT CAC TTA TTT 2475
811 Phe Leu Phe Leu Gln His Ile Ser Leu His Leu His His Leu Phe 825

2476 CTT TAT 2481
826 Leu Tyr

>gb|DS571171.1|:11243-13723 Entamoeba histolytica HM-1:IMSS scf_1104750517413 genomic scaffold, whole genome shotgun sequence Frame 2

2 GAG ATT TAA AAA ATA AAT GAT ATG ATG TCT AAA TAA AAA TAG AAA 46
1 Glu Ile End Lys Ile Asn Asp Met Met Ser Lys End Lys End Lys 15

47 ATT GAT ATT TTA TTT TTA TGA AAA TAA CGA GTA TAA TCT ATG AGA 91

16 Ile Asp Ile Leu Phe Leu End Lys End Arg Val End Ser Met Arg 30

92 TTA TTC ATT TCA TTA TTG TCA ATT TTA TTA TTA ATG TCA AAC CAC 136
31 Leu Phe Ile Ser Leu Leu Ser Ile Leu Leu Leu Met Ser Asn His 45

137 ACT TTA TGA CAG AAG AAG TAA TTT TTT CAT TAA ATT AAA CTA ATG 181
46 Thr Leu End Gln Lys Lys End Phe Phe His End Ile Lys Leu Met 60

182 TTA AAT CCT CGA TAA ATT AAT TTT TAA TTT CCT AAG TCA ATA TAA 226
61 Leu Asn Pro Arg End Ile Asn Phe End Phe Pro Lys Ser Ile End 75

227 TAA ATA TTC TTC ATT TTG GTG ATA TGA GAA GAA ATT ATA CAT TTT 271
76 End Ile Phe Phe Ile Leu Val Ile End Glu Glu Ile Ile His Phe 90

272 TTA AGA AGA TTG GTA AAC GCC ATT ATG TGA TGA ATA GTA CAT TTT 316
91 Leu Arg Arg Leu Val Asn Ala Ile Met End End Ile Val His Phe 105

317 AAA ACT TTA TGT CTT TCA ATT TCT GTT AGT CAG TTA AGT GAA ACA 361
106 Lys Thr Leu Cys Leu Ser Ile Ser Val Ser Gln Leu Ser Glu Thr 120

362 ATG TTA TTT TAT AAA TTA ATA TTA TTT TCT ATA AGA TAT TGC CAT 406
121 Met Leu Phe Tyr Lys Leu Ile Leu Phe Ser Ile Arg Tyr Cys His 135

407 ATT AAT AAA CAA TGG AAT TTT CAT TTT ATT GAA CTT TAA GAG AAC 451
136 Ile Asn Lys Gln Trp Asn Phe His Phe Ile Glu Leu End Glu Asn 150

452 AAA GAT TAA TTA GAG TAT ATG TAT TTA AAA GAC TAA ATG TTG GTG 496
151 Lys Asp End Leu Glu Tyr Met Tyr Leu Lys Asp End Met Leu Val 165

497 GTA AGA GTT TTT TAG AAA AGA GTT AAT TAA CTC AAA GGT ATT GAA 541
166 Val Arg Val Phe End Lys Arg Val Asn End Leu Lys Gly Ile Glu 180

542 GAA AGA AGT GTT TTG AAT AAC TTC ATT GTA TTG GTA GTT AAT TAA 586
181 Glu Arg Ser Val Leu Asn Asn Phe Ile Val Leu Val Val Asn End 195

587 AAT GAA ATA AAA GAC TCT CGT TAT AAG TAG GAA GAT GAA GAG CAA 631
196 Asn Glu Ile Lys Asp Ser Arg Tyr Lys End Glu Asp Glu Glu Gln 210

632 CAT TCA TTA GAA AAA TTG ATT TCA TTG CAT AAT ATT ATT TAT TGA 676
211 His Ser Leu Glu Lys Leu Ile Ser Leu His Asn Ile Ile Tyr End 225

677 ATA ATT TTT TAA TTC TTC TAT CAT AAA AAA TAC AAA AAA ATG AAT 721
226 Ile Ile Phe End Phe Phe Tyr His Lys Lys Tyr Lys Lys Met Asn 240

722 AAA TTC AAT AAT ATA ATA AAT CTC ATT TAG AAA AAG TAT AGT GCA
766
241 Lys Phe Asn Asn Ile Ile Asn Leu Ile End Lys Lys Tyr Ser Ala 255

767 GGA ACA AAA CGA GTA TTT CCA TTA AAA CCA ACA AAT GTC TGT GGT
811
256 Gly Thr Lys Arg Val Phe Pro Leu Lys Pro Thr Asn Val Cys Gly 270

812 TGT ATC ACT AAT TCT CTA CAC ATA GGA CAT CTT GGT TGT TGA ATA
856
271 Cys Ile Thr Asn Ser Leu His Ile Gly His Leu Gly Cys End Ile 285

857 AGC CAT TTA AAA ATA CAT TCT TCA CAA TAA ACA TGA CCA CAT TTT
901
286 Ser His Leu Lys Ile His Ser Ser Gln End Thr End Pro His Phe 300

902 AAC TTA ATT GGA CGA TTA ACT GTG TCT TGA CAA ATT AAG CAC ATT
946
301 Asn Leu Ile Gly Arg Leu Thr Val Ser End Gln Ile Lys His Ile 315

947 TTG TCT TCT AAT GAA TCT ACG GTT ACT GAA GTA CCA AAC ATA TTG
991
316 Leu Ser Ser Asn Glu Ser Thr Val Thr Glu Val Pro Asn Ile Leu 330

992 TTT TGA GTG AAA AAG GTT TTA AGA ACT TTA AAG AAA CAA GAA AGA
1036
331 Phe End Val Lys Lys Val Leu Arg Thr Leu Lys Lys Gln Glu Arg 345

1037 AAA TTA ACA AAT CCA AGA CAA CGT TCA AAC ACA TAA AGC CAC GTT
1081
346 Lys Leu Thr Asn Pro Arg Gln Arg Ser Asn Thr End Ser His Val 360

1082 ATT GGA TAT ATA ACA ATA TAA CTA ATC CAT TGG TTG GTT ATT GAA
1126
361 Ile Gly Tyr Ile Thr Ile End Leu Ile His Trp Leu Val Ile Glu 375

1127 GTG TAT GAA ATA AGA TAA TTA TAA ATA ATA AAA GTA GGA AGA ATA
1171
376 Val Tyr Glu Ile Arg End Leu End Ile Ile Lys Val Gly Arg Ile 390

1172 ACT CTT ATA GAC AAA GTA AGT GAT TCT ATC ATA GAG AGT AAT TTA 1216
391 Thr Leu Ile Asp Lys Val Ser Asp Ser Ile Ile Glu Ser Asn Leu 405

1217 CAT AAT AAT AAT ATA TTT TTA ACT AAA AAT ATT GTT AAA ACT TTT 1261
406 His Asn Asn Asn Ile Phe Leu Thr Lys Asn Ile Val Lys Thr Phe 420

1262 AGA TAA TAA TTT ATA TAA CGG TAA ATA CAG TCA CAT AGA TAT AAA 1306
421 Arg End End Phe Ile End Arg End Ile Gln Ser His Arg Tyr Lys 435

1307 TAA ATG AAT AAA TCA CTA AAA GAA TTT GGA ATA ACA GTA ATA TCT 1351
436 End Met Asn Lys Ser Leu Lys Glu Phe Gly Ile Thr Val Ile Ser 450

1352 ATC ATA TCA GTT ATT CCT ATT TGC CAC ACC TTT ATA ATA AAT AAT 1396
451 Ile Ile Ser Val Ile Pro Ile Cys His Thr Phe Ile Ile Asn Asn 465

1397 AAC ATA GAA GGA ATA CAA TAA ACT AAT ATC CTT ATT TTA TTT TCC 1441
466 Asn Ile Glu Gly Ile Gln End Thr Asn Ile Leu Ile Leu Phe Ser 480

1442 CCA GTA TTT ACA TAT TGT GTA ACT AAC TCA GTT AAA TGA TAA TGT 1486
481 Pro Val Phe Thr Tyr Cys Val Thr Asn Ser Val Lys End End Cys 495

1487 TGA ATG AAA TCA ACT ATA ATA AAT AAA GTA TAA AAA ATA TGA CTA 1531
496 End Met Lys Ser Thr Ile Ile Asn Lys Val End Lys Ile End Leu 510

1532 AAG AAA AAG AAT AAT ATA ACT ACT ATA TAA GAC CCA AAA CCT GAA 1576
511 Lys Lys Lys Asn Asn Ile Thr Thr Ile End Asp Pro Lys Pro Glu 525

1577 TCA TAA TCA TTA TTT ATA TAA TCA TTA TCA CCA TCA TTA TTA TTA 1621
526 Ser End Ser Leu Phe Ile End Ser Leu Ser Pro Ser Leu Leu Leu 540

1622 TTA TTT TGG TTG TTT GGT TGT TGT TCA TTT CCT GTA TGA GGT ACT 1666
541 Leu Phe Trp Leu Phe Gly Cys Cys Ser Phe Pro Val End Gly Thr 555

1667 TCA TTT AAT TGA GTT AAA CCT TCT TCT CTC AAA TTA ACT TCA TGT 1711

556 Ser Phe Asn End Val Lys Pro Ser Ser Leu Lys Leu Thr Ser Cys 570

1712 TCA TCA ACA TTT CCC ATT TCA ATT TCG TGT TCA AAT TTT GTG TCT 1756
571 Ser Ser Thr Phe Pro Ile Ser Ile Ser Cys Ser Asn Phe Val Ser 585

1757 TGG TTT TCA TTA GTA TTT AAT GAA TTT ATA TGA TTA TTT TCA TGA 1801
586 Trp Phe Ser Leu Val Phe Asn Glu Phe Ile End Leu Phe Ser End 600

1802 GTG TCT ATA CAA CTC AAT TTA TTT GTA CAG ACA ATA TCA CTA TCA 1846
601 Val Ser Ile Gln Leu Asn Leu Phe Val Gln Thr Ile Ser Leu Ser 615

1847 CAT TCT TTT TCT GTT TTC TGT TTA TTA AGA TGT TCT TCA GTT TGT 1891
616 His Ser Phe Ser Val Phe Cys Leu Leu Arg Cys Ser Ser Val Cys 630

1892 GAC ATT TAA GTG ATT TTA TAG TAT TTT TTT GTA AAC TTT TTA GAA 1936
631 Asp Ile End Val Ile Leu End Tyr Phe Phe Val Asn Phe Leu Glu 645

1937 ATA AAA AAT GAG CAG AAA CAG AAT ATC AAA AAC CCT TTA TTA TTT 1981
646 Ile Lys Asn Glu Gln Lys Gln Asn Ile Lys Asn Pro Leu Leu Phe 660

1982 ATT CTT AAT CAA ATC AAC CAT CTC CTC AAT TGT TAT CTC ATT AAA 2026
661 Ile Leu Asn Gln Ile Asn His Leu Leu Asn Cys Tyr Leu Ile Lys 675

2027 TGT GTG AAT TGC CAG GTT GCA TCC TTT AAT TTG AAG TAA TTC ATT 2071
676 Cys Val Asn Cys Gln Val Ala Ser Phe Asn Leu Lys End Phe Ile 690

2072 AAC AGT CAT TTC TGA GGC AAT TCC TAT TGG AGT AGC ACC AGC TGA 2116
691 Asn Ser His Phe End Gly Asn Ser Tyr Trp Ser Ser Thr Ser End 705

2117 TAA TGC AGA ATG AAT ACC TGA AAT AGT ATC TTC AAA TAC AAT ACA 2161
706 End Cys Arg Met Asn Thr End Asn Ser Ile Phe Lys Tyr Asn Thr 720

2162 ATG AGA AAT ACT AAC TCC TAA TTT CTT AGA AGC TTT TAA ATA AAT 2206
721 Met Arg Asn Thr Asn Ser End Phe Leu Arg Ser Phe End Ile Asn 735

2207 GTC AGG AGC AGG TTT ACC TTT AAA AGT AAA ATC ATT AAA AAT AAT 2251
736 Val Arg Ser Arg Phe Thr Phe Lys Ser Lys Ile Ile Lys Asn Asn 750

2252 TTT ATC AAT ATC AAA CCA TTC TTC CAA GTG GTA TTT TTG AAT AAA 2296
751 Phe Ile Asn Ile Lys Pro Phe Phe Gln Val Val Phe Leu Asn Lys 765

2297 CAC TTG AAC ATT TCC CCA ATC ACT TGA AGT GGC AAT AGT AAA AGG 2341
766 His Leu Asn Ile Ser Pro Ile Thr End Ser Gly Asn Ser Lys Arg 780

2342 AAT ATT ACA TTT TTT TAA TGC CTC AAA AAG ATT AAT TGC ACC ATC 2386
781 Asn Ile Thr Phe Phe End Cys Leu Lys Lys Ile Asn Cys Thr Ile 795

2387 ACA TAA TTT TAT ATC ACT TTT CAT TAA CAT AGT TCT ATA TAA ATT 2431
796 Thr End Phe Tyr Ile Thr Phe His End His Ser Ser Ile End Ile 810

2432 TTC TTT TTC TTC AGC ATA TTT CTT TGC ATC TTC ATC ACT TAT TTC 2476
811 Phe Phe Phe Phe Ser Ile Phe Leu Cys Ile Phe Ile Thr Tyr Phe 825

2477 TTT 2479
826 Phe 826

>gb|DS571171.1|:11243-13723 Entamoeba histolytica HM-1:IMSS scf_1104750517413 genomic scaffold, whole genome shotgun sequence Frame 3

3 AGA TTT AAA AAA TAA ATG ATA TGA TGT CTA AAT AAA AAT AGA AAA 47
0 Arg Phe Lys Lys End Met Ile End Cys Leu Asn Lys Asn Arg Lys 14

48 TTG ATA TTT TAT TTT TAT GAA AAT AAC GAG TAT AAT CTA TGA GAT 92
15 Leu Ile Phe Tyr Phe Tyr Glu Asn Asn Glu Tyr Asn Leu End Asp 29

93 TAT TCA TTT CAT TAT TGT CAA TTT TAT TAT TAA TGT CAA ACC ACA 137
30 Tyr Ser Phe His Tyr Cys Gln Phe Tyr Tyr End Cys Gln Thr Thr 44

138 CTT TAT GAC AGA AGA AGT AAT TTT TTC ATT AAA TTA AAC TAA TGT 182
45 Leu Tyr Asp Arg Arg Ser Asn Phe Phe Ile Lys Leu Asn End Cys 59

183 TAA ATC CTC GAT AAA TTA ATT TTT AAT TTC CTA AGT CAA TAT AAT 227
60 End Ile Leu Asp Lys Leu Ile Phe Asn Phe Leu Ser Gln Tyr Asn 74

228 AAA TAT TCT TCA TTT TGG TGA TAT GAG AAG AAA TTA TAC ATT TTT 272
75 Lys Tyr Ser Ser Phe Trp End Tyr Glu Lys Lys Leu Tyr Ile Phe 89

273 TAA GAA GAT TGG TAA ACG CCA TTA TGT GAT GAA TAG TAC ATT TTA 317
90 End Glu Asp Trp End Thr Pro Leu Cys Asp Glu End Tyr Ile Leu 104

318 AAA CTT TAT GTC TTT CAA TTT CTG TTA GTC AGT TAA GTG AAA CAA 362
105 Lys Leu Tyr Val Phe Gln Phe Leu Leu Val Ser End Val Lys Gln 119

363 TGT TAT TTT ATA AAT TAA TAT TAT TTT CTA TAA GAT ATT GCC ATA 407
120 Cys Tyr Phe Ile Asn End Tyr Tyr Phe Leu End Asp Ile Ala Ile 134

408 TTA ATA AAC AAT GGA ATT TTC ATT TTA TTG AAC TTT AAG AGA ACA 452
135 Leu Ile Asn Asn Gly Ile Phe Ile Leu Leu Asn Phe Lys Arg Thr 149

453 AAG ATT AAT TAG AGT ATA TGT ATT TAA AAG ACT AAA TGT TGG TGG 497
150 Lys Ile Asn End Ser Ile Cys Ile End Lys Thr Lys Cys Trp Trp 164

498 TAA GAG TTT TTT AGA AAA GAG TTA ATT AAC TCA AAG GTA TTG AAG 542
165 End Glu Phe Phe Arg Lys Glu Leu Ile Asn Ser Lys Val Leu Lys 179

543 AAA GAA GTG TTT TGA ATA ACT TCA TTG TAT TGG TAG TTA ATT AAA 587
180 Lys Glu Val Phe End Ile Thr Ser Leu Tyr Trp End Leu Ile Lys 194

588 ATG AAA TAA AAG ACT CTC GTT ATA AGT AGG AAG ATG AAG AGC AAC 632
195 Met Lys End Lys Thr Leu Val Ile Ser Arg Lys Met Lys Ser Asn 209

633 ATT CAT TAG AAA AAT TGA TTT CAT TGC ATA ATA TTA TTT ATT GAA 677
210 Ile His End Lys Asn End Phe His Cys Ile Ile Leu Phe Ile Glu 224

678 TAA TTT TTT AAT TCT TCT ATC ATA AAA AAT ACA AAA AAA TGA ATA 722
225 End Phe Phe Asn Ser Ser Ile Ile Lys Asn Thr Lys Lys End Ile 239

723 AAT TCA ATA ATA TAA TAA ATC TCA TTT AGA AAA AGT ATA GTG CAG 767
240 Asn Ser Ile Ile End End Ile Ser Phe Arg Lys Ser Ile Val Gln 254

768 GAA CAA AAC GAG TAT TTC CAT TAA AAC CAA CAA ATG TCT GTG GTT 812
255 Glu Gln Asn Glu Tyr Phe His End Asn Gln Gln Met Ser Val Val 269

813 GTA TCA CTA ATT CTC TAC ACA TAG GAC ATC TTG GTT GTT GAA TAA 857
270 Val Ser Leu Ile Leu Tyr Thr End Asp Ile Leu Val Val Glu End 284

858 GCC ATT TAA AAA TAC ATT CTT CAC AAT AAA CAT GAC CAC ATT TTA 902
285 Ala Ile End Lys Tyr Ile Leu His Asn Lys His Asp His Ile Leu 299

903 ACT TAA TTG GAC GAT TAA CTG TGT CTT GAC AAA TTA AGC ACA TTT 947
300 Thr End Leu Asp Asp End Leu Cys Leu Asp Lys Leu Ser Thr Phe 314

948 TGT CTT CTA ATG AAT CTA CGG TTA CTG AAG TAC CAA ACA TAT TGT 992
315 Cys Leu Leu Met Asn Leu Arg Leu Leu Lys Tyr Gln Thr Tyr Cys 329

993 TTT GAG TGA AAA AGG TTT TAA GAA CTT TAA AGA AAC AAG AAA GAA 1037
330 Phe Glu End Lys Arg Phe End Glu Leu End Arg Asn Lys Lys Glu 344

1038 AAT TAA CAA ATC CAA GAC AAC GTT CAA ACA CAT AAA GCC ACG TTA 1082
345 Asn End Gln Ile Gln Asp Asn Val Gln Thr His Lys Ala Thr Leu 359

1083 TTG GAT ATA TAA CAA TAT AAC TAA TCC ATT GGT TGG TTA TTG AAG 1127
360 Leu Asp Ile End Gln Tyr Asn End Ser Ile Gly Trp Leu Leu Lys 374

1128 TGT ATG AAA TAA GAT AAT TAT AAA TAA TAA AAG TAG GAA GAA TAA 1172
375 Cys Met Lys End Asp Asn Tyr Lys End End Lys End Glu Glu End 389

1173 CTC TTA TAG ACA AAG TAA GTG ATT CTA TCA TAG AGA GTA ATT TAC 1217
390 Leu Leu End Thr Lys End Val Ile Leu Ser End Arg Val Ile Tyr 404

1218 ATA ATA ATA ATA TAT TTT TAA CTA AAA ATA TTG TTA AAA CTT TTA 1262
405 Ile Ile Ile Ile Tyr Phe End Leu Lys Ile Leu Leu Lys Leu Leu 419

1263 GAT AAT AAT TTA TAT AAC GGT AAA TAC AGT CAC ATA GAT ATA AAT 1307
420 Asp Asn Asn Leu Tyr Asn Gly Lys Tyr Ser His Ile Asp Ile Asn 434

1308 AAA TGA ATA AAT CAC TAA AAG AAT TTG GAA TAA CAG TAA TAT CTA 1352

435 Lys End Ile Asn His End Lys Asn Leu Glu End Gln End Tyr Leu 449

1353 TCA TAT CAG TTA TTC CTA TTT GCC ACA CCT TTA TAA TAA ATA ATA 1397
450 Ser Tyr Gln Leu Phe Leu Phe Ala Thr Pro Leu End End Ile Ile 464

1398 ACA TAG AAG GAA TAC AAT AAA CTA ATA TCC TTA TTT TAT TTT CCC 1442
465 Thr End Lys Glu Tyr Asn Lys Leu Ile Ser Leu Phe Tyr Phe Pro 479

1443 CAG TAT TTA CAT ATT GTG TAA CTA ACT CAG TTA AAT GAT AAT GTT 1487
480 Gln Tyr Leu His Ile Val End Leu Thr Gln Leu Asn Asp Asn Val 494

1488 GAA TGA AAT CAA CTA TAA TAA ATA AAG TAT AAA AAA TAT GAC TAA 1532
495 Glu End Asn Gln Leu End End Ile Lys Tyr Lys Lys Tyr Asp End 509

1533 AGA AAA AGA ATA ATA TAA CTA CTA TAT AAG ACC CAA AAC CTG AAT 1577
510 Arg Lys Arg Ile Ile End Leu Leu Tyr Lys Thr Gln Asn Leu Asn 524

1578 CAT AAT CAT TAT TTA TAT AAT CAT TAT CAC CAT CAT TAT TAT TAT 1622
525 His Asn His Tyr Leu Tyr Asn His Tyr His His His Tyr Tyr Tyr 539

1623 TAT TTT GGT TGT TTG GTT GTT GTT CAT TTC CTG TAT GAG GTA CTT 1667
540 Tyr Phe Gly Cys Leu Val Val Val His Phe Leu Tyr Glu Val Leu 554

1668 CAT TTA ATT GAG TTA AAC CTT CTT CTC TCA AAT TAA CTT CAT GTT 1712
555 His Leu Ile Glu Leu Asn Leu Leu Leu Ser Asn End Leu His Val 569

1713 CAT CAA CAT TTC CCA TTT CAA TTT CGT GTT CAA ATT TTG TGT CTT 1757
570 His Gln His Phe Pro Phe Gln Phe Arg Val Gln Ile Leu Cys Leu 584

1758 GGT TTT CAT TAG TAT TTA ATG AAT TTA TAT GAT TAT TTT CAT GAG 1802
585 Gly Phe His End Tyr Leu Met Asn Leu Tyr Asp Tyr Phe His Glu 599

1803 TGT CTA TAC AAC TCA ATT TAT TTG TAC AGA CAA TAT CAC TAT CAC 1847
600 Cys Leu Tyr Asn Ser Ile Tyr Leu Tyr Arg Gln Tyr His Tyr His 614

1848 ATT CTT TTT CTG TTT TCT GTT TAT TAA GAT GTT CTT CAG TTT GTG 1892
615 Ile Leu Phe Leu Phe Ser Val Tyr End Asp Val Leu Gln Phe Val 629

1893 ACA TTT AAG TGA TTT TAT AGT ATT TTT TTG TAA ACT TTT TAG AAA 1937
630 Thr Phe Lys End Phe Tyr Ser Ile Phe Leu End Thr Phe End Lys 644

1938 TAA AAA ATG AGC AGA AAC AGA ATA TCA AAA ACC CTT TAT TAT TTA 1982
645 End Lys Met Ser Arg Asn Arg Ile Ser Lys Thr Leu Tyr Tyr Leu 659

1983 TTC TTA ATC AAA TCA ACC ATC TCC TCA ATT GTT ATC TCA TTA AAT 2027
660 Phe Leu Ile Lys Ser Thr Ile Ser Ser Ile Val Ile Ser Leu Asn 674

2028 GTG TGA ATT GCC AGG TTG CAT CCT TTA ATT TGA AGT AAT TCA TTA 2072
675 Val End Ile Ala Arg Leu His Pro Leu Ile End Ser Asn Ser Leu 689

2073 ACA GTC ATT TCT GAG GCA ATT CCT ATT GGA GTA GCA CCA GCT GAT 2117
690 Thr Val Ile Ser Glu Ala Ile Pro Ile Gly Val Ala Pro Ala Asp 704

2118 AAT GCA GAA TGA ATA CCT GAA ATA GTA TCT TCA AAT ACA ATA CAA 2162
705 Asn Ala Glu End Ile Pro Glu Ile Val Ser Ser Asn Thr Ile Gln 719

2163 TGA GAA ATA CTA ACT CCT AAT TTC TTA GAA GCT TTT AAA TAA ATG 2207
720 End Glu Ile Leu Thr Pro Asn Phe Leu Glu Ala Phe Lys End Met 734

2208 TCA GGA GCA GGT TTA CCT TTA AAA GTA AAA TCA TTA AAA ATA ATT 2252
735 Ser Gly Ala Gly Leu Pro Leu Lys Val Lys Ser Leu Lys Ile Ile 749

2253 TTA TCA ATA TCA AAC CAT TCT TCC AAG TGG TAT TTT TGA ATA AAC 2297
750 Leu Ser Ile Ser Asn His Ser Ser Lys Trp Tyr Phe End Ile Asn 764

2298 ACT TGA ACA TTT CCC CAA TCA CTT GAA GTG GCA ATA GTA AAA GGA 2342
765 Thr End Thr Phe Pro Gln Ser Leu Glu Val Ala Ile Val Lys Gly 779

2343 ATA TTA CAT TTT TTT AAT GCC TCA AAA AGA TTA ATT GCA CCA TCA 2387

780 Ile Leu His Phe Phe Asn Ala Ser Lys Arg Leu Ile Ala Pro Ser 794

2388 CAT AAT TTT ATA TCA CTT TTC ATT AAC ATA GTT CTA TAT AAA TTT 2432
795 His Asn Phe Ile Ser Leu Phe Ile Asn Ile Val Leu Tyr Lys Phe 809

2433 TCT TTT TCT TCA GCA TAT TTC TTT GCA TCT TCA TCA CTT ATT TCT 2477
810 Ser Phe Ser Ser Ala Tyr Phe Phe Ala Ser Ser Ser Leu Ile Ser 824

2478 TTA 2480
825 Leu 825

Figure S1: Translated nucleotide of the EhslncRNA transcript. Diagrammatic representation of all the three frames of EhslncRNA transcript translated using Bioedit program to look for ORFs in the gene. All the three frames displayed multiple stop codons (End shown in red) in the transcript. Each codon is read as left nucleotide, top nucleotide and right nucleotide.

```
                                       10        20        30        40        50        60        70
gb|DS571171.1|:11243-13723    AGAGATTTAAAAAATAAATGATATGATGTCTAAATAAAAATAGAAAATTGATATTTTATTTTTATGAAAATAACGAG
EhRm_scaffold00669            AGAGATTTAAAAAATAAATGATATGATGTCTAAATAAAAATAGAAAATTGATATTTTATTTTTATGAAAATAACGAG
tmp.EHDS4_3292                AGAGATTTAAAAAATAAATGATATGATGTCTAAATAAAAATAGAAAATTGATATTTTATTTTTATGAAAATAACGAG
tmp.EHMS96_3292               AGAGATTTAAAAAATAAATGATATGATGTCTAAATAAAAATAGAAAATTGATATTTTATTTTTATGAAAATAACGAG
KB638462                      AGAGATTTAAAAAATAAATGATATGATGTCTAAATAAAAATAGAAAATTGATATTTTATTTTTATGAAAATAACGAG
KB444973                      AGAGATTTAAAAAATAAATGATATGATGTCTAAATAAAAATAGAAAATTGATATTTTATTTTTATGAAAATAACGAG
KB823609                      AGAGATTTAAAAAATAAATGATATGATGTCTAAATAAAAATAGAAAATTGATATTTTATTTTTATGAAAATAACGAG
KB611040                      AGAGATTTAAAAAATAAATGATATGATGTCTAAATAAAAATAGAAAATTGATATTTTATTTTTATGAAAATAACGAG
tmp.EHKU48_3292               -----------------------------------------------------------------------------
tmp.EHKU50_3292               -----------------------------------------------------------------------------
tmp.EHHM1_CA_3292             -----------------------------------------------------------------------------
Clustal Consensus
```

```
                                      110       120       130       140       150       160       170
gb|DS571171.1|:11243-13723    TCATTATTGTCAATTTTATTATTAATGTCAAACCACACTTTATGACAGAAGAAGTAATTTTTTCATTAAATTAAACT
EhRm_scaffold00669            TCATTATTGTCAATTTTATTATTAATGTCAAACCACACTTTATGACAGAAGAAGTAATTTTTTCATTAAATTAAACT
tmp.EHDS4_3292                TCATTATTGTCAATTTTATTATTAATGTCAAACCACACTTTATGACAGAAGAAGTAATTTTTTCATTAAATTAAACT
tmp.EHMS96_3292               TCATTATTGTCAATTTTATTATTAATGTCAAACCACACTTTATGACAGAAGAAGTAATTTTTTCATTAAATTAAACT
KB638462                      TCATTATTGTCAATTTTATTATTAATGTCAAACCACACTTTATGACAGAAGAAGTAATTTTTTCATTAAATTAAACT
KB444973                      TCATTATTGTCAATTTTATTATTAATGTCAAACCACACTTTATGACAGAAGAAGTAATTTTTTCATTAAATTAAACT
KB823609                      TCATTATTGTCAATTTTATTATTAATGTCAAACCACACTTTATGACAGAAGAAGTAATTTTTTCATTAAATTAAACT
KB611040                      TCATTATTGTCAATTTTATTATTAATGTCAAACCACACTTTATGACAGAAGAAGTAATTTTTTCATTAAATTAAACT
tmp.EHKU48_3292               -----------------------------------------------------------------------------
tmp.EHKU50_3292               -----------------------------------------------------------------------------
tmp.EHHM1_CA_3292             -----------------------------------------------------------------------------
Clustal Consensus
```

```
                                      210       220       230       240       250       260       270
gb|DS571171.1|:11243-13723    ATTTTTAATTTCCTAAGTCAATATAATAAATATTCTTCATTTTGGTGATATGAGAAGAAATTATACATTTTTTAAGA
EhRm_scaffold00669            ATTTTTAATTTCCTAAGTCAATATAATAAATATTCTTCATTTTGGTGATATGAGAAGAAATTATACATTTTTTAAGA
tmp.EHDS4_3292                ATTTTTAATTTCCTAAGTCAATATAATAAATATTCTTCATTTTGGTGATATGAGAAGAAATTATACATTTTTTAAGA
tmp.EHMS96_3292               ATTTTTAATTTCCTAAGTCAATATAATAAATATTCTTCATTTTGGTGATATGAGAAGAAATTATACATTTTTTAAGA
KB638462                      ATTTTTAATTTCCTAAGTCAATATAATAAATATTCTTCATTTTGGTGATATGAGAAGAAATTATACATTTTTTAAGA
KB444973                      ATTTTTAATTTCCTAAGTCAATATAATAAATATTCTTCATTTTGGTGATATGAGAAGAAATTATACATTTTTTAAGA
KB823609                      ATTTTTAATTTCCTAAGTCAATATAATAAATATTCTTCATTTTGGTGATATGAGAAGAAATTATACATTTTTTAAGA
KB611040                      ATTTTTAATTTCCTAAGTCAATATAATAAATATTCTTCATTTTGGTGATATGAGAAGAAATTATACATTTTTTAAGA
tmp.EHKU48_3292               -----------------------------------------------------------------------------
tmp.EHKU50_3292               -----------------------------------------------------------------------------
tmp.EHHM1_CA_3292             -----------------------------------------------------------------------------
Clustal Consensus
```

```
                                      310       320       330       340       350       360       370
gb|DS571171.1|:11243-13723    ATGAATAGTACATTTTAAAACTTTATGTCTTTCAATTTCTGTTAGTCAGTTAAGTGAAACAATGTTATTTTATAAAT
EhRm_scaffold00669            ATGAATAGTACATTTTAAAACTTTATGTCTTTCAATTTCTGTTAGTCAGTTAAGTGAAACAATGTTATTTTATAAAT
tmp.EHDS4_3292                ATGAATAGTACATTTTAAAACTTTATGTCTTTCAATTTCTGTTAGTCAGTTAAGTGAAACAATGTTATTTTATAAAT
tmp.EHMS96_3292               ATGAATAGTACATTTTAAAACTTTATGTCTTTCAATTTCTGTTAGTCAGTTAAGTGAAACAATGTTATTTTATAAAT
KB638462                      ATGAATAGTACATTTTAAAACTTTATGTCTTTCAATTTCTGTTAGTCAGTTAAGTGAAACAATGTTATTTTATAAAT
KB444973                      ATGAATAGTACATTTTAAAACTTTATGTCTTTCAATTTCTGTTAGTCAGTTAAGTGAAACAATGTTATTTTATAAAT
KB823609                      ATGAATAGTACATTTTAAAACTTTATGTCTTTCAATTTCTGTTAGTCAGTTAAGTGAAACAATGTTATTTTATAAAT
KB611040                      ATGAATAGTACATTTTAAAACTTTATGTCTTTCAATTTCTGTTAGTCAGTTAAGTGAAACAATGTTATTTTATAAAT
tmp.EHKU48_3292               -----------------------------------------------------------------------------
tmp.EHKU50_3292               -----------------------------------------------------------------------------
tmp.EHHM1_CA_3292             -----------------------------------------------------------------------------
Clustal Consensus
```

```
                                      410       420       430       440       450       460       470
gb|DS571171.1|:11243-13723    TGCCATATTAATAAACAATGGAATTTTCATTTTATTGAACTTTAAGAGAACAAAGATTAATTAGAGTATATGTATTT
EhRm_scaffold00669            TGCCATATTAATAAACAATGGAATTTTCATTTTATTGAACTTTAAGAGAACAAAGATTAATTAGAGTATATGTATTT
tmp.EHDS4_3292                TGCCATATTAATAAACAATGGAATTTTCATTTTATTGAACTTTAAGAGAACAAAGATTAATTAGAGTATATGTATTT
tmp.EHMS96_3292               TGCCATATTAATAAACAATGGAATTTTCATTTTATTGAACTTTAAGAGAACAAAGATTAATTAGAGTATATGTATTT
KB638462                      TGCCATATTAATAAACAATGGAATTTTCATTTTATTGAACTTTAAGAGAACAAAGATTAATTAGAGTATATGTATTT
KB444973                      TGCCATATTAATAAACAATGGAATTTTCATTTTATTGAACTTTAAGAGAACAAAGATTAATTAGAGTATATGTATTT
KB823609                      TGCCATATTAATAAACAATGGAATTTTCATTTTATTGAACTTTAAGAGAACAAAGATTAATTAGAGTATATGTATTT
KB611040                      TGCCATATTAATAAACAATGGAATTTTCATTTTATTGAACTTTAAGAGAACAAAGATTAATTAGAGTATATGTATTT
tmp.EHKU48_3292               -----------------------------------------------------------------------------
tmp.EHKU50_3292               -----------------------------------------------------------------------------
tmp.EHHM1_CA_3292             -----------------------------------------------------------------------------
Clustal Consensus
```

```
                                      510       520       530       540       550       560       570
gb|DS571171.1|:11243-13723    GAGTTTTTTAGAAAAGAGTTAATTAACTCAAAGGTATTGAAGAAAGAAGTGTTTTGAATAACTTCATTGTATTGGTA
EhRm_scaffold00669            GAGTTTTTTAGAAAAGAGTTAATTAACTCAAAGGTATTGAAGAAAGAAGTGTTTTGAATAACTTCATTGTATTGGTA
tmp.EHDS4_3292                GAGTTTTTTAGAAAAGAGTTAATTAACTCAAAGGTATTGAAGAAAGAAGTGTTTTGAATAACTTCATTGTATTGGTA
tmp.EHMS96_3292               GAGTTTTTTAGAAAAGAGTTAATTAACTCAAAGGTATTGAAGAAAGAAGTGTTTTGAATAACTTCATTGTATTGGTA
KB638462                      GAGTTTTTTAGAAAAGAGTTAATTAACTCAAAGGTATTGAAGAAAGAAGTGTTTTGAATAACTTCATTGTATTGGTA
KB444973                      GAGTTTTTTAGAAAAGAGTTAATTAACTCAAAGGTATTGAAGAAAGAAGTGTTTTGAATAACTTCATTGTATTGGTA
KB823609                      GAGTTTTTTAGAAAAGAGTTAATTAACTCAAAGGTATTGAAGAAAGAAGTGTTTTGAATAACTTCATTGTATTGGTA
KB611040                      GAGTTTTTTAGAAAAGAGTTAATTAACTCAAAGGTATTGAAGAAAGAAGTGTTTTGAATAACTTCATTGTATTGGTA
tmp.EHKU48_3292               -----------------------------------------------------------------------------
tmp.EHKU50_3292               -----------------------------------------------------------------------------
tmp.EHHM1_CA_3292             -----------------------------------------------------------------------------
Clustal Consensus
```

```
                                      610       620       630       640       650       660       670
gb|DS571171.1|:11243-13723    CTCTCGTTATAAGTAGGAAGATGAAGAGCAACATTCATTAGAAAAATTGATTTCATTGCATAATATTATTTATTGAA
EhRm_scaffold00669            CTCTCGTTATAAGTAGGAAGATGAAGAGCAACATTCATTAGAAAAATTGATTTCATTGCATAATATTATTTATTGAA
tmp.EHDS4_3292                CTCTCGTTATAAGTAGGAAGATGAAGAGCAACATTCATTAGAAAAATTGATTTCATTGCATAATATTATTTATTGAA
tmp.EHMS96_3292               CTCTCGTTATAAGTAGGAAGATGAAGAGCAACATTCATTAGAAAAATTGATTTCATTGCATAATATTATTTATTGAA
KB638462                      CTCTCGTTATAAGTAGGAAGATGAAGAGCAACATTCATTAGAAAAATTGATTTCATTGCATAATATTATTTATTGAA
KB444973                      CTCTCGTTATAAGTAGGAAGATGAAGAGCAACATTCATTAGAAAAATTGATTTCATTGCATAATATTATTTATTGAA
```

```
KB823609                       CTCTCGTTATAAGTAGGAAGATGAAGAGCAACATTCATTAGAAAAATTGATTTCATTGCATAATATTATTTATTGAA
KB611040                       CTCTCGTTATAAGTAGGAAGATGAAGAGCAACATTCATTAGAAAAATTGATTTCATTGCATAATATTATTTATTGAA
tmp.EHKU48_3292                -------------------------------------------------------------------------ATAA
tmp.EHKU50_3292                -----------------------------------------------------------------------------
tmp.EHHM1_CA_3292              -----------------------------------------------------------------------------
Clustal Consensus

                                        710       720       730       740       750       760       770
gb|DS571171.1|:11243-13723     AAAAAATACAAAAAAATGAATAAATTCAATAATATAATAAATCTCATTTAGAAAAAGTATAGTGCAGGAACAAAACG
EhRm_scaffold00669             AAAAAATACAAAAAAATGAATAAATTCAATAATATAATAAATCTCATTTAGAAAAAGTATAGTGCAGGAACAAAACG
tmp.EHDS4_3292                 AAAAAATACAAAAAAATGAATAAATTCAATAATATAATAAATCTCATTTAGAAAAAGTATAGTGCAGGAACAAAACG
tmp.EHMS96_3292                AAAAAATACAAAAAAATGAATAAATTCAATAATATAATAAATCTCATTTAGAAAAAGTATAGTGCAGGAACAAAACG
KB638462                       AAAAAATACAAAAAAATGAATAAATTCAATAATATAATAAATCTCATTTAGAAAAAGTATAGTGCAGGAACAAAACG
KB444973                       AAAAAATACAAAAAAATGAATAAATTCAATAATATAATAAATCTCATTTAGAAAAAGTATAGTGCAGGAACAAAACG
KB823609                       AAAAAATACAAAAAAATGAATAAATTCAATAATATAATAAATCTCATTTAGAAAAAGTATAGTGCAGGAACAAAACG
KB611040                       AAAAAATACAAAAAAATGAATAAATTCAATAATATAATAAATCTCATTTAGAAAAAGTATAGTGCAGGAACAAAACG
tmp.EHKU48_3292                AAAAATAACAAAAAAATGAATAAATTCAATAATATAATAAATCTCATTTAGAAAAAGTATAGTGCAGGAACAAAACG
tmp.EHKU50_3292                -----------------------------------------------------------------------------
tmp.EHHM1_CA_3292              -----------------------------------------------------------------------------
Clustal Consensus

                                        810       820       830       840       850       860       870
gb|DS571171.1|:11243-13723     AAATGTCTGTGGTTGTATCACTAATTCTCTACACATAGGACATCTTGGTTGTTGAATAAGCCATTTAAAAATACATT
EhRm_scaffold00669             AAATGTCTGTGGTTGTATCACTAATTCTCTACACATAGGACATCTTGGTTGTTGAATAAGCCATTTAAAAATACATT
tmp.EHDS4_3292                 AAATGTCTGTGGTTGTATCACTAATTCTCTACACATAGGACATCTTGGTTGTTGAATAAGCCATTTAAAAATACATT
tmp.EHMS96_3292                AAATGTCTGTGGTTGTATCACTAATTCTCTACACATAGGACATCTTGGTTGTTGAATAAGCCATTTAAAAATACATT
KB638462                       AAATGTCTGTGGTTGTATCACTAATTCTCTACACATAGGACATCTTGGTTGTTGAATAAGCCATTTAAAAATACATT
KB444973                       AAATGTCTGTGGTTGTATCACTAATTCTCTACACATAGGACATCTTGGTTGTTGAATAAGCCATTTAAAAATACATT
KB823609                       AAATGTCTGTGGTTGTATCACTAATTCTCTACACATAGGACATCTTGGTTGTTGAATAAGCCATTTAAAAATACATT
KB611040                       AAATGTCTGTGGTTGTATCACTAATTCTCTACACATAGGACATCTTGGTTGTTGAATAAGCCATTTAAAAATACATT
tmp.EHKU48_3292                AAATGTCTGTGGTTGTATCACTAATTCTCTACACATAGGACATCTTGGTTGTTGAATAAGCCATTTAAAAATACATT
tmp.EHKU50_3292                -----------------------------------------------------------------------------
tmp.EHHM1_CA_3292              -----------------------------------------------------------------------------
Clustal Consensus

                                        910       920       930       940       950       960       970
gb|DS571171.1|:11243-13723     TTTAACTTAATTGGACGATTAACTGTGTCTTGACAAATTAAGCACATTTTGTCTTCTAATGAATCTACGGTTACTGA
EhRm_scaffold00669             TTTAACTTAATTGGACGATTAACTGTGTCTTGACAAATTAAGCACATTTTGTCTTCTAATGAATCTACGGTTACTGA
tmp.EHDS4_3292                 TTTAACTTAATTGGACGATTAACTGTGTCTTGACAAATTAAGCACATTTTGTCTTCTAATGAATCTACGGTTACTGA
tmp.EHMS96_3292                TTTAACTTAATTGGACGATTAACTGTGTCTTGACAAATTAAGCACATTTTGTCTTCTAATGAATCTACGGTTACTGA
KB638462                       TTTAACTTAATTGGACGATTAACTGTGTCTTGACAAATTAAGCACATTTTGTCTTCTAATGAATCTACGGTTACTGA
KB444973                       TTTAACTTAATTGGACGATTAACTGTGTCTTGACAAATTAAGCACATTTTGTCTTCTAATGAATCTACGGTTACTGA
KB823609                       TTTAACTTAATTGGACGATTAACTGTGTCTTGACAAATTAAGCACATTTTGTCTTCTAATGAATCTACGGTTACTGA
KB611040                       TTTAACTTAATTGGACGATTAACTGTGTCTTGACAAATTAAGCACATTTTGTCTTCTAATGAATCTACGGTTACTGA
tmp.EHKU48_3292                TTTAACTTAATTGGACGATTAACTGTGTCTTGACAAATTAAGCACATTTTGTCTTCTAATGAATCTACGGTTACTGA
tmp.EHKU50_3292                ---------------------------------------------ATTTTGTCTTCTAATGAATCTACGGTTACTGA
tmp.EHHM1_CA_3292              -----------------------------------------------------------------------------
Clustal Consensus

                                        1010      1020      1030      1040      1050      1060      1070
gb|DS571171.1|:11243-13723     TGAAAAAGGTTTTAAGAACTTTAAAGAAACAAGAAAGAAAATTAACAAATCCAAGACAACGTTCAAACACATAAAGC
EhRm_scaffold00669             TGAAAAAGGTTTTAAGAACTTTAAAGAAACAAGAAAGAAAATTAACAAATCCAAGACAACGTTCAAACACATAAAGC
tmp.EHDS4_3292                 TGAAAAAGGTTTTAAGAACTTTAAAGAAACAAGAAAGAAAATTAACAAATCCAAGACAACGTTCAAACACATAAAGC
tmp.EHMS96_3292                TGAAAAAGGTTTTAAGAACTTTAAAGAAACAAGAAAGAAAATTAACAAATCCAAGACAACGTTCAAACACATAAAGC
KB638462                       TGAAAAAGGTTTTAAGAACTTTAAAGAAACAAGAAAGAAAATTAACAAATCCAAGACAACGTTCAAACACATAAAGC
KB444973                       TGAAAAAGGTTTTAAGAACTTTAAAGAAACAAGAAAGAAAATTAACAAATCCAAGACAACGTTCAAACACATAAAGC
KB823609                       TGAAAAAGGTTTTAAGAACTTTAAAGAAACAAGAAAGAAAATTAACAAATCCAAGACAACGTTCAAACACATAAAGC
KB611040                       TGAAAAAGGTTTTAAGAACTTTAAAGAAACAAGAAAGAAAATTAACAAATCCAAGACAACGTTCAAACACATAAAGC
tmp.EHKU48_3292                TGAAAAAGGTTTTAAGAACTTTAAAGAAACAAGAAAGAAAATTAACAAATCCAAGACAACGTTCAAACACATAAAGC
tmp.EHKU50_3292                TGAAAAAGGTTTTAAGAACTTTAAAGAAACAAGAAAGAAAATTAACAAATCCAAGACAACGTTCAAACACATAAAGC
tmp.EHHM1_CA_3292              -----------------------------------------------------------------------------
Clustal Consensus

                                        1110      1120      1130      1140      1150      1160      1170
gb|DS571171.1|:11243-13723     ATAACTAATCCATTGGTTGGTTATTGAAGTGTATGAAATAAGATAATTATAAATAATAAAAGTAGGAAGAATAACTC
EhRm_scaffold00669             ATAACTAATCCATTGGTTGGTTATTGAAGTGTATGAAATAAGATAATTATAAATAATAAAAGTAGGAAGAATAACTC
tmp.EHDS4_3292                 ATAACTAATCCATTGGTTGGTTATTGAAGTGTATGAAATAAGATAATTATAAATAATAAAAGTAGGAAGAATAACTC
tmp.EHMS96_3292                ATAACTAATCCATTGGTTGGTTATTGAAGTGTATGAAATAAGATAATTATAAATAATAAAAGTAGGAAGAATAACTC
KB638462                       ATAACTAATCCATTGGTTGGTTATTGAAGTGTATGAAATAAGATAATTATAAATAATAAAAGTAGGAAGAATAACTC
KB444973                       ATAACTAATCCATTGGTTGGTTATTGAAGTGTATGAAATAAGATAATTATAAATAATAAAAGTAGGAAGAATAACTC
KB823609                       ATAACTAATCCATTGGTTGGTTATTGAAGTGTATGAAATAAGATAATTATAAATAATAAAAGTAGGAAGAATAACTC
KB611040                       ATAACTAATCCATTGGTTGGTTATTGAAGTGTATGAAATAAGATAATTATAAATAATAAAAGTAGGAAGAATAACTC
tmp.EHKU48_3292                ATAACTAATCCATTGGTTGGTTATTGAAGTGTATGAAATAAGATAATTATAAATAATAAAAGTAGGAAGAATAACTC
tmp.EHKU50_3292                ATAACTAATCCATTGGTTGGTTATTGAAGTGTATGAAATAAGATAATTATAAATAATAAAAGTAGGAAGAATAACTC
tmp.EHHM1_CA_3292              -----------------------------------------------------------------------------
Clustal Consensus

                                        1210      1220      1230      1240      1250      1260      1270
gb|DS571171.1|:11243-13723     ATCATAGAGAGTAATTTACATAATAATAATATATTTTTAACTAAAAATATTGTTAAAACTTTTAGATAATAATTTAT
EhRm_scaffold00669             ATCATAGAGAGTAATTTACATAATAATAATATATTTTTAACTAAAAATATTGTTAAAACTTTTAGATAATAATTTAT
tmp.EHDS4_3292                 ATCATAGAGAGTAATTTACATAATAATAATATATTTTTAACTAAAAATATTGTTAAAACTTTTAGATAATAATTTAT
tmp.EHMS96_3292                ATCATAGAGAGTAATTTACATAATAATAATATATTTTTAACTAAAAATATTGTTAAAACTTTTAGATAATAATTTAT
KB638462                       ATCATAGAGAGTAATTTACATAATAATAATATATTTTTAACTAAAAATATTGTTAAAACTTTTAGATAATAATTTAT
KB444973                       ATCATAGAGAGTAATTTACATAATAATAATATATTTTTAACTAAAAATATTGTTAAAACTTTTAGATAATAATTTAT
KB823609                       ATCATAGAGAGTAATTTACATAATAATAATATATTTTTAACTAAAAATATTGTTAAAACTTTTAGATAATAATTTAT
KB611040                       ATCATAGAGAGTAATTTACATAATAATAATATATTTTTAACTAAAAATATTGTTAAAACTTTTAGATAATAATTTAT
tmp.EHKU48_3292                ATCATAGAGAGTAATTTACATAATAATAATATATTTTTAACTAAAAATATTGTTAAAACTTTTAGATAATAATT-AT
tmp.EHKU50_3292                ATCATAGAGAGTAATTTACATAATAATAATATATTTTTAACTAAAAATATTGTTAAAACTTTTAGATAATAATTTAT
tmp.EHHM1_CA_3292              -----------------------------------------------------------------------------
Clustal Consensus

                                        1310      1320      1330      1340      1350      1360      1370
```

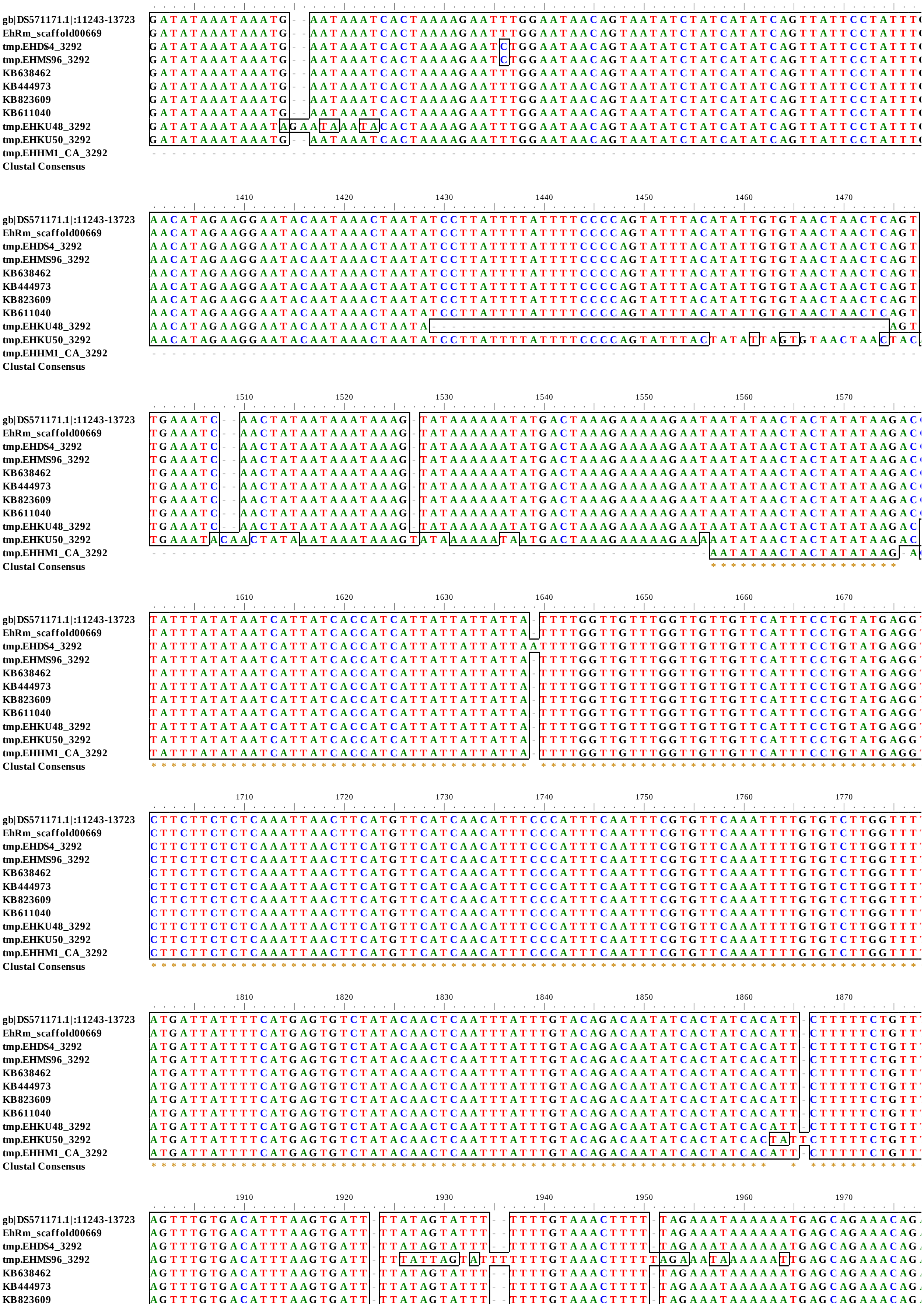

gb|DS571171.1|:11243-13723
EhRm_scaffold00669
tmp.EHDS4_3292
tmp.EHMS96_3292
KB638462
KB444973
KB823609
KB611040
tmp.EHKU48_3292
tmp.EHKU50_3292
tmp.EHHM1_CA_3292
Clustal Consensus
1410
1420
1430
1440
1450
1460
1470
1510
1520
1530
1540
1550
1560
1570
1610
1620
1630
1640
1650
1660
1670
1710
1720
1730
1740
1750
1760
1770
1810
1820
1830
1840
1850
1860
1870
1910
1920
1930
1940
1950
1960
1970

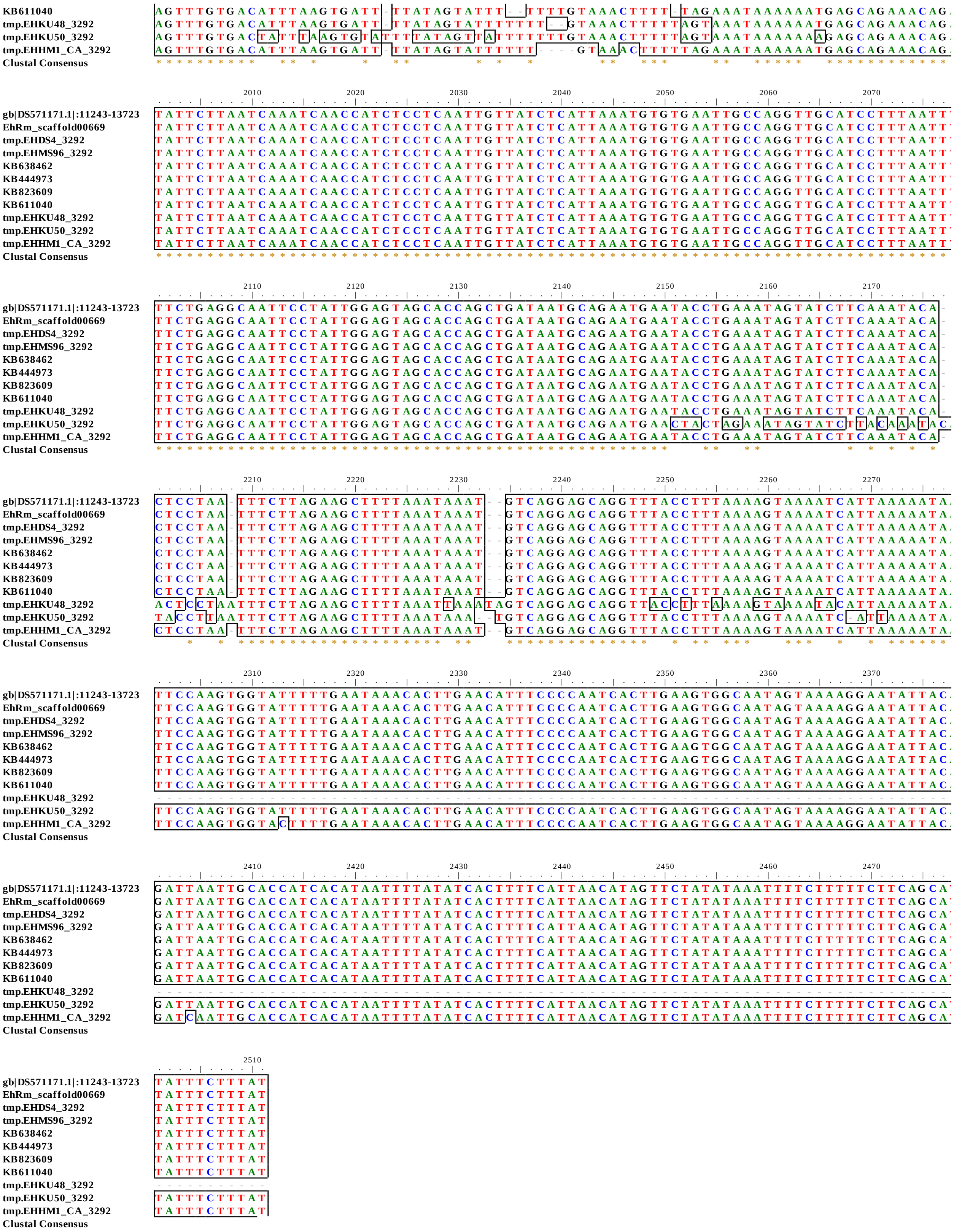
KB611040
tmp.EHKU48_3292
tmp.EHKU50_3292
tmp.EHHM1_CA_3292
Clustal Consensus
2010
2020
2030
2040
2050
2060
2070
gb|DS571171.1|:11243-13723
EhRm_scaffold00669
tmp.EHDS4_3292
tmp.EHMS96_3292
KB638462
KB444973
KB823609
KB611040
tmp.EHKU48_3292
tmp.EHKU50_3292
tmp.EHHM1_CA_3292
Clustal Consensus
2110
2120
2130
2140
2150
2160
2170
2210
2220
2230
2240
2250
2260
2270
2310
2320
2330
2340
2350
2360
2370
2410
2420
2430
2440
2450
2460
2470
2510

Figure S2. Multiple alignment of EhslncRNA gene sequence from *Entamoeba histolytica* HM1: IMSS with other strains of *Entamoeba histolytica*. Genome databases of different strains of *E. histolytica*; *E. histolytica* Rahman, *E. histolytica* DS4-868, *E. histolytica* MS96-3382, *E. histolytica* HM-3:IMSS, *E. histolytica* KU-27, *E. histolytica* HM-1:IMSS-A, *E. histolytica* HM1-:IMSS-B, *E. histolytica* KU-48, *E. histolytica* KU-50 , *E. histolytica* HM1-CA respectively available in amoebadb using EhslncRNA sequence as query. The figure shows the multiple alignment of all these sequences retrieved from the search and alignment done using freely available online multiple sequence alignment tool Clustal omega. The gene shows high level of conservation among the different strains of *E. histolytica* indicating its importance in the parasite. Conserved nucleotides are marked with asterisk. Identical nucleotides marked with boxes.

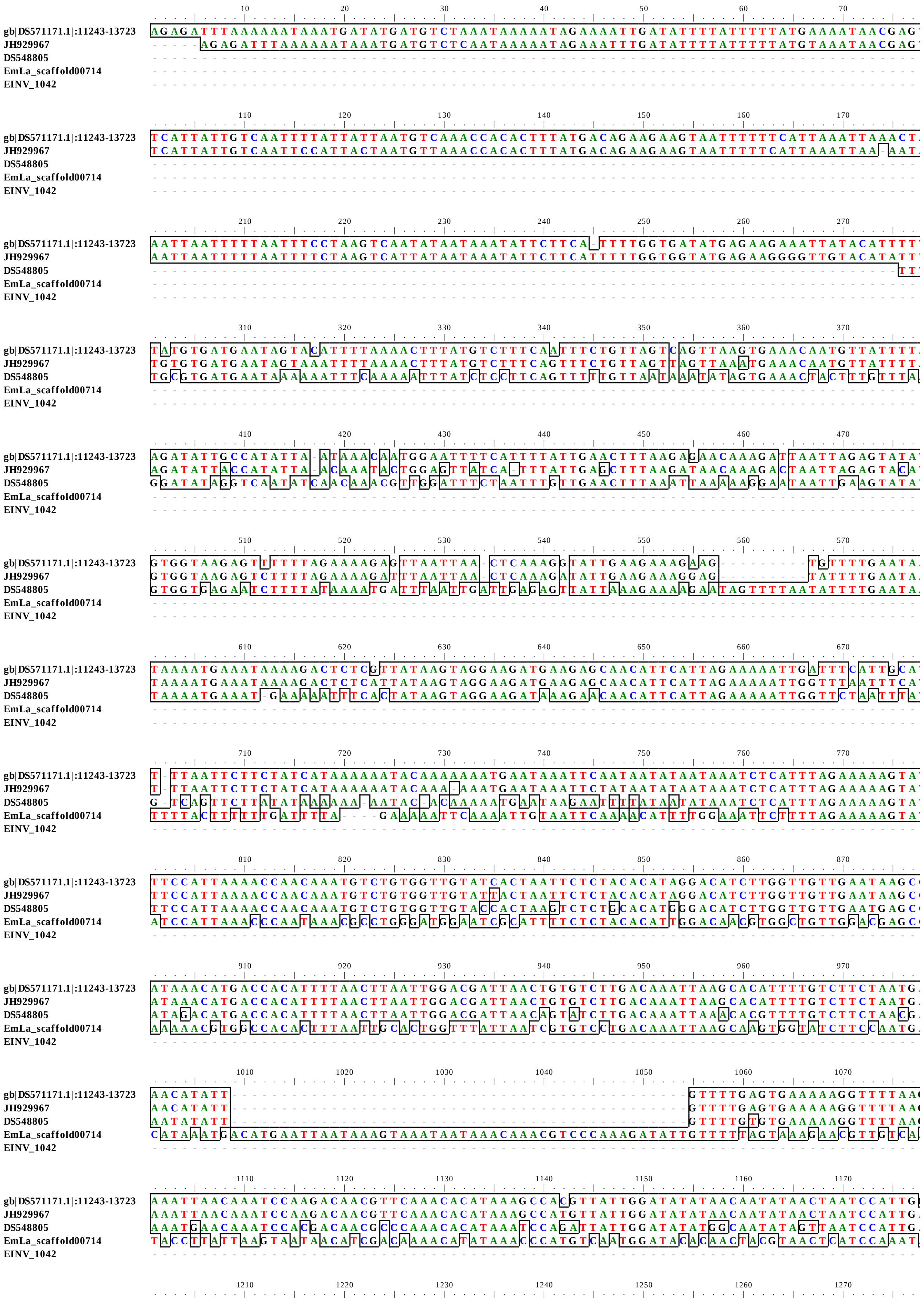
gb|DS571171.1|:11243-13723
JH929967
DS548805
EmLa_scaffold00714
EINV_1042

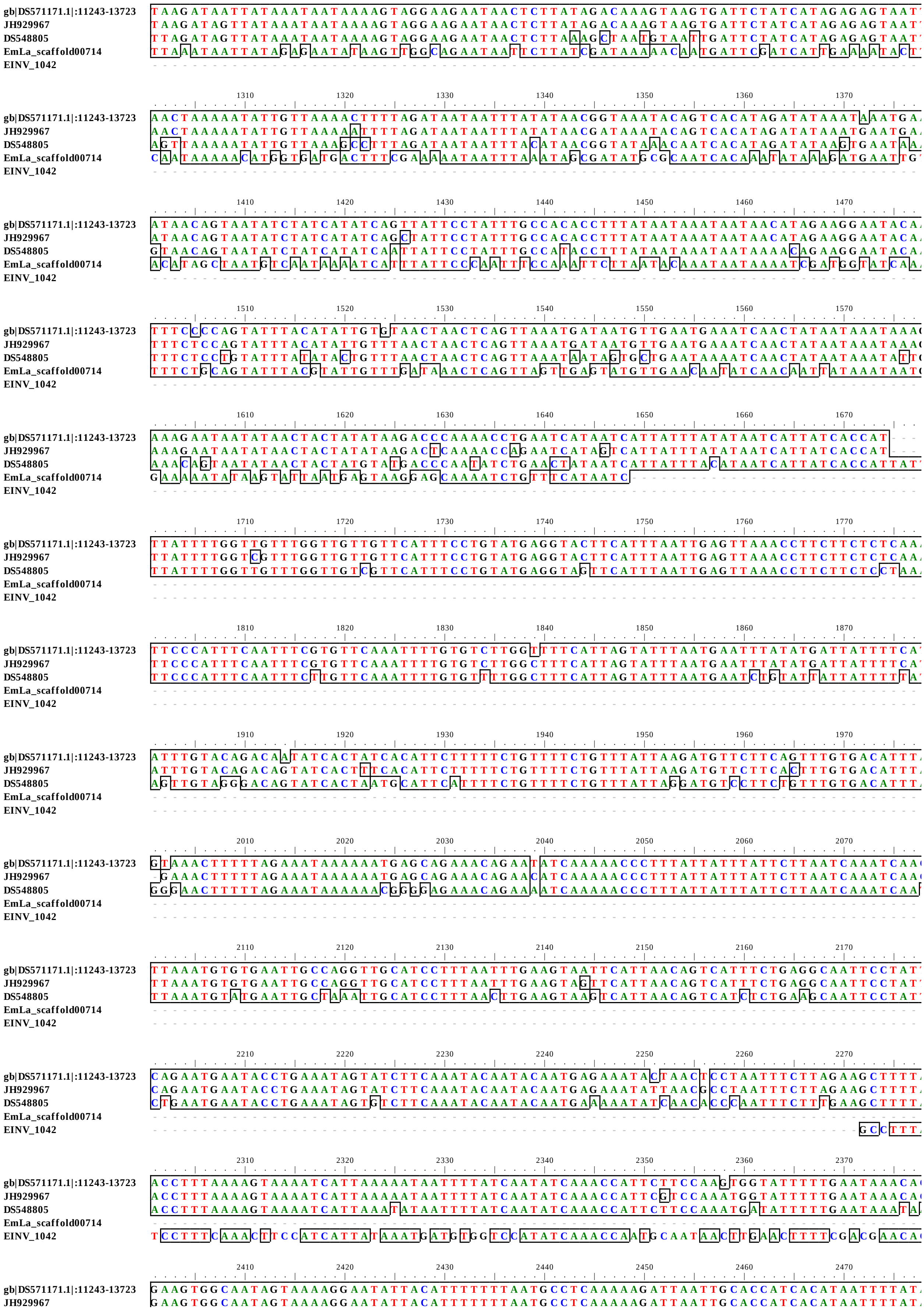

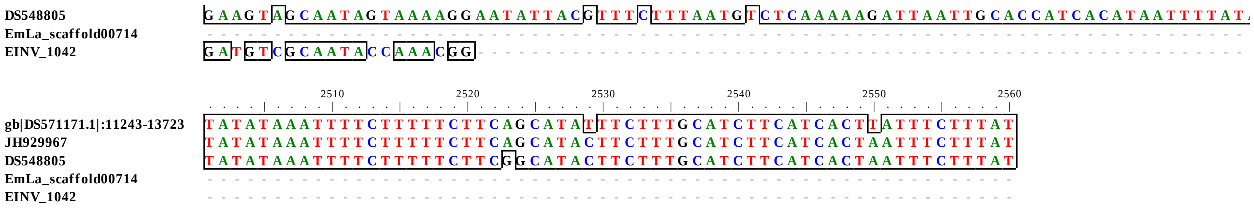

Figure S3. Multiple alignment of EhslncRNA gene with other species of *Entamoeba*. Genome databases of *E. nutalli* P19, *E. dispar* SAW 760, *E. moshkovskii* Laredo, *E. invadens* IP1 respectively were searched using EhslncRNA gene sequence as query in amoebadb. The sequences were retrieved from the search and multiple alignment was done using Clustal omega tool. The figure shows multiple alignment of the sequences of all the above mentioned *Entamoeba* species. The sequence shows high level of similarity across the whole sequence in *E. nutalli* and *E. dispar*. Identical nucleotides are represented by boxes.

Table S1. List of primers used in the study for cloning in p-Eh-Neo-Luc shuttle vector

**Name of the oligo**	**Primer sequence(5'-3')**
EhslncR-437 FP	CGGCTCGAGAAATTAATGAAATTAGAGAAATTAGG
EhslncR-45 RP	CGCGGGTACCAATTAGTTTATTCATACTTAT
EhslncR-33 RP	GCGGGTACCATACTTATGGTTTGAATATG
EhslncR-118FP	CGGCTCGAGCTTCAAGACAATGATGGTTTAC
EhslncR-346FP	CGGCTCGAGCGAAATATAGCATAGTCTGATGAC

Annealing temperature - 48ºC

Table S2. List of cloning done in the present study

**Name of the clone**	**Insert**	**Size of the insert (kb)**	**Vector**	**Size of the vector(kb)**	**Cloning sites**	**Primer pair**
EhslncR Pr 1	EhslncR Pr483	0.483	p-Eh-NEO-LUC-S	7.8	*Xho I* and *Kpn I*	EhslncR-437 FP &EhslncR-45 RP
EhslncR Pr 2	EhslncR Pr470	0.470	p-Eh-NEO-LUC-S	7.8	*Xho I* and *Kpn I*	EhslncR-437 FP & EhslncR-33RP
EhslncR Pr 3	EhslncR Pr391	0.391	p-Eh-NEO-LUC-S	7.8	*Xho I* and *Kpn I*	EhslncR-346FP & EhslncR-45RP
EhT18 Pr 4	EhslncR 163	0.163	p-Eh-NEO-LUC-S	7.8	*Xho I* and *Kpn I*	EhslncR-118FP & EhslncR-45RP
